# Glial subtype-specific modulation of disease pathogenesis in *Drosophila* models of ALS

**DOI:** 10.1016/j.gendis.2025.101631

**Published:** 2025-04-08

**Authors:** Yanan Wei, Hayong Rhee, Hadi Najafi, Shane Blair, Nam Chul Kim, Woo Jae Kim

**Affiliations:** a The HIT Center for Life Sciences, Harbin Institute of Technology, Harbin, Heilongjiang 150080, China; b Department of Cellular and Molecular Medicine, University of Ottawa, Ottawa, K1H 8M5, Canada; c Department of Molecular, Cell and Cancer Biology, University of Massachusetts Chan Medical School, Worcester, MA 01655, USA; d Department of Pharmacy Practice and Pharmaceutical Sciences, College of Pharmacy, University of Minnesota, Duluth, MN 55812, USA; e Medical and Health Research Institute, Zhengzhou Research Institute of HIT, Zhengzhou, Henan 450044, China

Amyotrophic lateral sclerosis (ALS) is a progressive neurodegenerative disorder whose mechanisms underlying remain incompletely understood, particularly the role of glial cells. This study investigated the impact of ALS-associated genes on distinct glial populations in *Drosophila*. We assessed motor function and lifespan, revealing significant sexual dimorphism, with males generally showing greater declines. Our findings also underscore the importance of glial cells, particularly subperineurial glia (SPG) in the male leg femur, and provides valuable insights into the complex interplay between glial cells and ALS-associated genes.

We utilized *nSyb*-GAL4 for pan-neuronal and *repo*-GAL4 for pan-glial expression. To investigate subtype-specific effects, we targeted six glial subtypes: cortex glia (CG), astrocyte-like glia (ALG), tract ensheathing glia (EGT), ensheathing glia (EGN), subperineurial (SPG) and perineurial glia (PNG).^1^ We assessed motor deficits and life expectancy, using climbing and lifespan assays.

Mutations in the SOD1 gene are associated with ALS1. In *Drosophila*, wild-type human SOD1 under *nSyb-GAL4* and *repo-GAL4* showed no effect on climbing or lifespan in young and old flies, except for a decline in climbing ability in older males with glial expression (Supplementary data 1A-F). FlySCope data indicate strong Sod1 expression in PNG and SPG glia (Supplementary data 1G). While glial expression in young males did not affect climbing (Fig. A1), it had no significant effect on females (Supplementary data 1H-I). However, 20-day-old males with SOD1 overexpression in ALG and EGN showed reduced climbing ability (Fig. A2), and male lifespan was shortened with expression in ALG, CG, PNG, and SPG (Fig. A3-5). Distinct phenotypes were observed in the hSOD1-G85R and hSOD1-A4V mutants (Supplementary Data 2-3). Notably, the hSOD1-A4V mutants exhibited greater toxicity when expressed in glia compared to other variants. This suggests that specific glial subtypes may play a critical role in mediating the detrimental effects of these mutants on survival and motor function.

**Figure 1:**
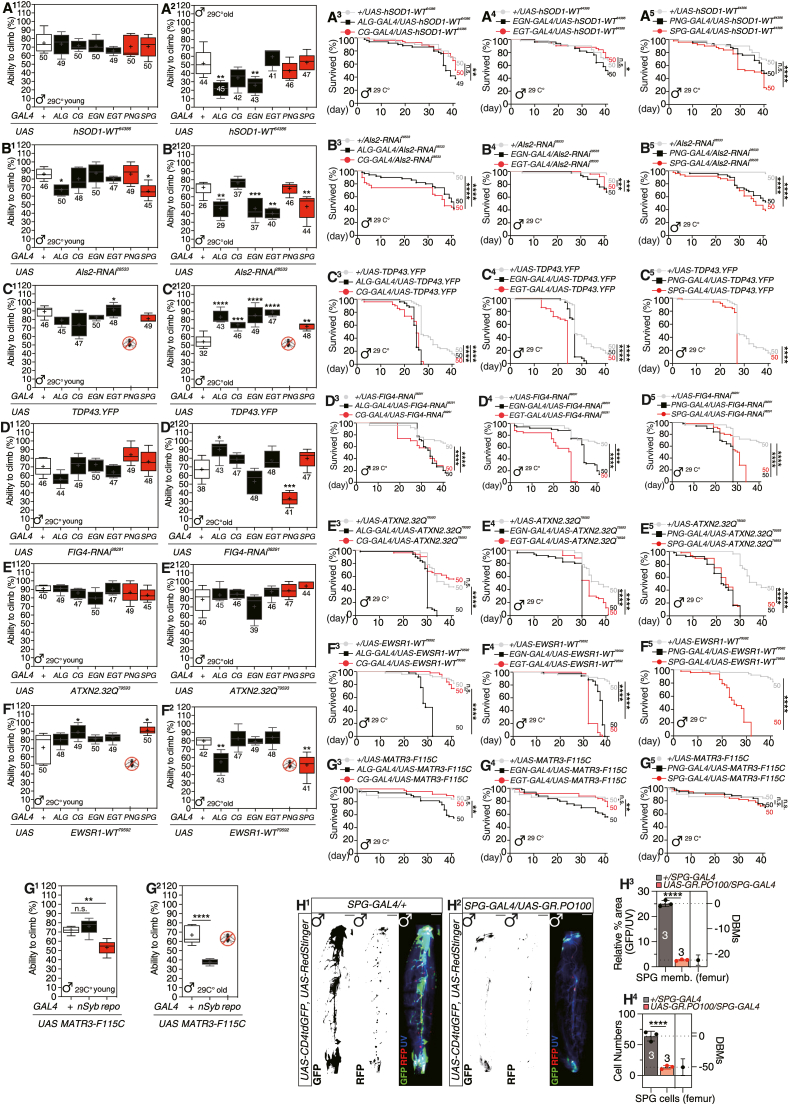
Screening of various ALS-related genes in glial and neuronal expression and femur immunostaining. **(A)** Climbing test of flies expressing SOD1-WT by subtype glial drivers, 5-day-old (A1) and 20-day-old males (A2). Lifespan of flies expressing SOD1-WT by subtype glial drivers, ALG and CG drivers (A3), EGN and EGT drivers (A4), PNG and SPG drivers (A5). **(B)** Climbing test of flies expressing Als2-RNAi2 by subtype glial drivers, 5-day-old (B1) and 20-day-old males (B2). Lifespan of flies expressing Als2-RNAi2 by subtype glial drivers, ALG and CG drivers (B3), EGN and EGT drivers (B4), PNG and SPG drivers (B5). **(C)** Climbing test of flies expressing TDP43.YFP by subtype glial drivers, 5-day-old (C1) and 20-day-old males (C2). Lifespan of flies expressing TDP43.YFP by subtype glial drivers, ALG and CG drivers (C3), EGN and EGT drivers (C4), PNG and SPG drivers (C5). **(D)** Climbing test of flies expressing FIG4-RNAi by subtype glial drivers, 5-day-old (D1) and 20-day-old males (D2). Lifespan of flies expressing FIG4-RNAi by subtype glial drivers, ALG and CG drivers (D3), EGN and EGT drivers (D4), PNG and SPG drivers (D5). **(E)** Climbing test of flies expressing ATXN2.32Q by subtype glial drivers, 5-day-old (E1) and 20-day-old males (E2). Lifespan of flies expressing ATXN2.32Q by subtype glial drivers, ALG and CG drivers (E3), EGN and EGT drivers (E4), PNG and SPG drivers (E5). **(F)** Climbing test of flies expressing EWSR1-WT by subtype glial drivers, 5-day-old (F1) and 20-day-old males (F2). Lifespan of flies expressing EWSR1-WT by subtype glial drivers, ALG and CG drivers (F3), EGN and EGT drivers (F4), PNG and SPG drivers (F5). **(G)** Climbing test of flies expressing MATR3-F115C by subtype glial drivers, 5-day-old (G1) and 20-day-old males (G2). Lifespan of flies expressing MATR3-F115C by subtype glial drivers, ALG and CG drivers (G3), EGN and EGT drivers (G4), PNG and SPG drivers (G5). **(H)** Proportion of surface glial cells between control and adult forelegs expressing *C9orf72* gene. (H1-2) The distribution of SPG in male foreleg femur region expressing *UAS-CD4GFP*, *UAS-RedStinger* together with *SPG-GAL4* and merged (H1). The distribution of SPG in male foreleg femur region expressing *UAS-CD4GFP*, *UAS-RedStinger* and *UAS-GR.PO100* together with *SPG-GAL4* and merged (H2). Scale bars represent 50 mm. (H3-4) The relative area (H3) and the cell numbers (H4) of SPG in male foreleg femur region.

*Drosophila*'s ALS2 gene, encoding *Alsin*, was investigated using Als2-RNAi. While Als2 deficiency didn't affect female neurons, it generally impaired fly survival and climbing ability (Supplementary Data 4A-F). scRNA-seq revealed Als2 expression in specific surface glia (Supplementary Data 4G). In 20-day-old males, Als2 depletion in ALG, EGN, EGT, and SPG glia reduced climbing ability, without effects in CG or PNG (Fig. B1-2). Lifespan was shortened in males with Als2 reduction in ALG, CG, PNG, and SPG (Fig. B3-5 and Supplementary Data 4H-I). Thus, Als2 expression in certain glial subtypes is essential for adult locomotion and lifespan.

ALS10, caused by TARDBP mutations, is associated with adult mortality due to neuronal TDP-43 overexpression. In *Drosophila*, glial TDP43-YFP expression impairs climbing and lifespan (Supplementary Data 5A-F), while expression in PNG glia causing developmental lethality in both sexes (Fig. C1-2, Supplementary Data 5H-I). Overexpression in other glial subtypes, except PNG, does not affect locomotion, while expression in SPG shortens lifespan (Fig. C3-5). RNAi-mediated knockdown of the *Drosophila* TDP-43 homolog, TBPH, in neurons or glia shows no significant role for glial TBPH in survival (Supplementary Data 6-7). In mice, the overexpression of TDP-43-Q331K in glial cells leads to impaired climbing ability and reduced lifespan in both sexes, a phenotype that has also been observed in *Drosophila* (Supplementary Data 8). In summary, wild-type expression of TDP-43 and knockdown of TBPH are more toxic to neurons, while the expression of the Q331 mutant mimics the glial toxicity that has been reported in mice.

ALS11, associated with a FIG4 (Phosphoinositide 5-ph*osphatase)* gene mutation, is linked to the human FIG4 homolog. Using RNAi, we observed that neuronal FIG4 downregulation significantly shortened lifespan within 20 days, while glial FIG4 reduction mildly affected it (Supplementary Data 9-10). Glial FIG4 expression was confirmed by scRNAseq dataset analysis (Supplementary Data 9G). Specifically, PNG glial FIG4 knockdown affected climbing in older males (Fig. D1-2), and knockdown across glial subtypes reduced adult lifespan (Fig. D3-5). These findings suggest that PNG is pivotal in FIG4-mediated ALS pathology in *Drosophila*.

A recent study linked intermediate length polyglutamine (polyQ) repeats in ATXN2, associated with ALS13, to ALS risk.^2^ In *Drosophila*, glial expression of ATXN2-32Q impairs climbing and reduces lifespan (Supplementary Data 11A-F), with the human ATXN2 ortholog, *Atx2*, being highly expressed in glia, especially surface glia (Supplementary Data 11G). While glial subtype-specific ATXN2-32Q expression minimally affects climbing (Fig. E1-2), it generally shortens lifespan, except in ALG (Fig. E3-5). These findings suggest that glial ATXN2 expression contributes to neurodegenerative phenotypes in ALS.

*EWSR1 (EWS RNA-BINDING PROTEIN 1)*, implicated in ALS pathogenesis, showed increased mortality when overexpressed in *Drosophila* glia and neurons (Supplementary Data 12A-F). The human *EWSR1* and *FUS* ortholog, *caz*, is notably expressed in glial cells, especially surface glia (Supplementary Data 12G). EWSR1 expression in PNG was lethal, while in SPG it affected older males' climbing ability (Fig. F1-2). Its expression in most glial subtypes reduced adult lifespan, except in ALG (Fig. F3-5). These findings suggest that EWSR1 expression in surface glia significantly contributes to ALS phenotype development in the fly model.

Mutations in Matr3 are associated with ALS and Spinal Muscular Atrophy (SMA).^3^ Lifespan is unaffected by MATR3-WT in neurons or glia expression (Supplementary Data 13A-B). While MATR3-S85C neuronal expression is benign, glial expression impairs climbing in older flies (Supplementary Data 13C-G). Some patients initially had S85C mutation were subsequently reclassified as having novel mutations, including T622A and F115C5. Glial expression of MATR3-F115C, as opposed to that of females, induces significant impairments in climbing and lifespan, with older males exhibiting a lethal phenotype (Fig. G1-2, Supplementary Data 14), suggesting a glial dysfunction link to the F115C mutation. Subtype glia screening identified CG, EGT, PNG, and SPG as key in MATR3-F115C toxicity (Fig. G3-5). MATR3-T622A expression causes climbing defects in older flies with minimal effect on lifespan (Supplementary Data 13H-L). These findings highlight glia's role in the ALS-associated F115C mutation's phenotypic expression in *Drosophila*.

The traditional neuron-centric view of ALS is being supplanted by evidence of non-neuronal cell involvement in disease pathogenesis, including neuroinflammatory activation and immune response alterations.^4^ The blood–brain barrier (BBB), formed by insect surface glia, plays a key role in ALS by potentially facilitating immune cell infiltration into the CNS.^5^ This underscores the importance of surface glia in understanding non-cell autonomous ALS mechanisms.

Despite acknowledged limitations, fly legs and wings offer several advantages for ALS modeling. These include a high glial-to-neuron ratio, integration of the peripheral nervous system, and the ability to directly observe glia–neuron interactions in both sexes (Supplementary Data 15-20). Our data indicate that the SPG in the male leg femur is an ideal platform for evaluating gene toxicity (Supplementary Data 21-22).

To validate this, we used SPG-GAL4 to express C9orf72's polyGR and analyzed SPG membrane coverage in the male foreleg femur with tub-GAL80ts (Fig. H1-2). We found a significant decrease in SPG membrane coverage and cell numbers compared to controls (Fig. H3-4), indicating that male leg SPG in our fly model is a representative model for ALS pathogenesis. The sexual dimorphism observed between male and female leg SPG presents an intriguing opportunity to investigate the role of sex differences in ALS.

In summary, our results suggest a link between male-specific SPG morphology in the leg femur and heightened ALS gene expression susceptibility, though the precise mechanism is unclear (Table .1). Further research is needed to identify the factors behind this sexual dimorphism, which is essential for enhancing ALS understanding and developing inclusive treatment strategies.

**Table 1:** Summary for lifespan and climbing assay phenotypes.

Gene	nSyb-GAL4			repo-GAL4			ALG-GAL4			CG-GAL4			EGN-GAL4			EGT-GAL4			PNG-GAL4			SPG-GAL4		
	Climbing		Lifespan	Climbing		Lifespan	Climbing		Lifespan	Climbing		Lifespan	Climbing		Lifespan	Climbing		Lifespan	Climbing		Lifespan	Climbing		Lifespan
♀ SOD-WT	N	N	N	N	N	N	N	+	N/A	N	N	N/A	N	N	N/A	N	+	N/A	N	N	N/A	N	+	N/A
♀ SOD-G85R	N	N	+	N	N	N	N	N	N/A	N	N	N/A	–	N	N/A	N	+	N/A	N	N	N/A	N	N	N/A
♀ SOD-A4V	N	–	–	N	–	–	N	N	N/A	N	N	N/A	N	N	N/A	N	N	N/A	N	N	N/A	N	N	N/A
♀ ALS2-RNAi	N	–	N	N	N	–	N	–	N/A	N	N	N/A	N	–	N/A	N	–	N/A	N	N	N/A	N	–	N/A
♀ TDP-43.YFP	N/A	N/A	N/A	N	–	–	N	N	N/A	N	N	N/A	N	N	N/A	N	+	N/A	N	N/A	N/A	N	N	N/A
♀ TDP-43.Q331K	–	–	N	N	N	–	N	N	N/A	N	N	N/A	N	N	N/A	–	–	N/A	N	N	N/A	N	N	N/A
♀ TBPH-RNAiˆ29517	N	N	N	N	N	–	N	+	N/A	+	+	N/A	+	+	N/A	+	+	N/A	+	+	N/A	+	+	N/A
♀ TBPH-RNAiˆ39014	N	N	N	N	N	–	N	N	N/A	N	+	N/A	N	N	N/A	N	+	N/A	N	N	N/A	N	N	N/A
♀ FIG4-RNAiˆ58063	N	N	–	N	N	N	N	N	N/A	N	+	N/A	N	N	N/A	N	N	N/A	N	N	N/A	+	N	N/A
♀ FIG4-RNAiˆ38291	–	N/A	–	–	N	N	N	N	N/A	N	+	N/A	–	N	N/A	N	N	N/A	N	N	N/A	N	N	N/A
♀ ATXN2.32Q	N	N	N	N	–	–	N	N	N/A	N	N	N/A	N	–	N/A	N	N	N/A	N	N	N/A	N	N	N/A
♀ EWSR1-WT	N/A	N/A	N/A	N/A	N/A	N/A	N	–	N/A	N	N	N/A	N	+	N/A	N	N	N/A	N/A	N/A	N/A	N	N	N/A
♀ MATR3-WT	N/A	N/A	N	N/A	N/A	N	N/A	N/A	N/A	N/A	N/A	N/A	N/A	N/A	N/A	N/A	N/A	N/A	N/A	N/A	N/A	N/A	N/A	N/A
♀ MATR3-S85C	+	N	N/A	N	–	N/A	N/A	N/A	N/A	N/A	N/A	N/A	N/A	N/A	N/A	N/A	N/A	N/A	N/A	N/A	N/A	N/A	N/A	N/A
♀ MATR3-T622A	–	N/A	N/A	–	–	N/A	N/A	N/A	N/A	N/A	N/A	N/A	N/A	N/A	N/A	N/A	N/A	N/A	N/A	N/A	N/A	N/A	N/A	N/A
♀ MATR3-F115C	N	+	–	N	+	N	N/A	N/A	N/A	N/A	N/A	N/A	N/A	N/A	N/A	N/A	N/A	N/A	N/A	N/A	N/A	N/A	N/A	N/A
♂ SOD-WT	N	N	N	N	–	N	N	–	–	N	N	N	N	–	–	N	N	N	N	N	N	N	N	–
♂ SOD-G85R	N	–	+	N	–	N	N	N	N	N	N	+	N	N	–	N	N	N	+	N	N	N	N	–
♂ SOD-A4V	N	–	–	N	–	–	N	N	–	N	N	N	N	N	–	N	N	–	N	N	–	N	N	–
♂ ALS2-RNAi	–	–	–	–	N	–	–	–	–	N	N	–	N	–	–	N	–	–	N	N	–	–	–	–
♂ TDP-43.YFP	N/A	N/A	N/A	–	–	–	N	+	–	N	+	–	N	+	–	+	+	–	N/A	N/A	N/A	N	+	–
♂ TDP-43.Q331K	N	–	–	+	N	N	+	N	–	N	N	–	+	+	–	N	+	N	+	N	N	N	N	–
♂ TBPH-RNAiˆ29517	N	N	–	N	N	–	+	+	–	+	+	–	+	+	N	+	+	–	+	+	–	+	+	–
♂ TBPH-RNAiˆ39014	+	N	–	–	–	–	N	N	–	N	N	–	N	N	–	N	N	–	N	N	–	N	N	–
♂ FIG4-RNAiˆ58063	N	–	–	N	–	–	N	–	N	N	N	N	+	–	–	N	–	–	+	N	–	+	N	–
♂ FIG4-RNAiˆ38291	–	N/A	–	–	N	–	N	+	–	N	N	–	N	N	–	N	N	–	N	–	–	N	N	–
♂ ATXN2.32Q	–	–	–	N	–	–	N	N	–	N	N	N	N	N	–	N	N	–	N	N	–	N	N	–
♂ EWSR1-WT	N/A	N/A	N/A	N/A	N/A	N/A	N	–	–	+	N	N	N	N	–	N	N	–	N/A	N/A	N/A	+	–	–
♂ MATR3-WT	N/A	N/A	–	N/A	N/A	N	N/A	N/A	N/A	N/A	N/A	N/A	N/A	N/A	N/A	N/A	N/A	N/A	N/A	N/A	N/A	N/A	N/A	N/A
♂ MATR3-S85C	–	N	N	–	–	N	N/A	N/A	N/A	N/A	N/A	N/A	N/A	N/A	N/A	N/A	N/A	N/A	N/A	N/A	N/A	N/A	N/A	N/A
♂ MATR3-T622A	N	–	N	N	–	–	N/A	N/A	N/A	N/A	N/A	N/A	N/A	N/A	N/A	N/A	N/A	N/A	N/A	N/A	N/A	N/A	N/A	N/A
♂ MATR3-F115C	N	–	N	–	N/A	–	N/A	N/A	–	N/A	N/A	N	N/A	N/A	–	N/A	N/A	N	N/A	N/A	N	N/A	N/A	N

The symbols in the table represent the observed effect: N (No effect), + (Positive effect), - (Negative effect), or N/A (No clear information available). Climbing Assay: Results for climbing are presented separately for flies aged 5 days (first column) and 20 days (second column).

## CRediT authorship contribution statement

**Yanan Wei:** Writing – review & editing, Visualization, Methodology. **Hayong Rhee:** Visualization, Methodology. **Hadi Najafi:** Visualization, Methodology, Conceptualization. **Shane Blair:** Visualization, Data curation. **Nam Chul Kim:** Writing – original draft, Supervision, Project administration, Formal analysis, Conceptualization. **Woo Jae Kim:** Writing – review & editing, Writing – original draft, Visualization, Validation, Supervision, Resources, Project administration, Methodology, Investigation, Funding acquisition, Formal analysis, Data curation, Conceptualization.

## Funding

This work was supported by University of Ottawa Startup grant to WJK, University of Ottawa Brain and Mind Research Institute/Center for Neural Dynamics Open call project grant to WJK, University of Ottawa Interdisciplinary Research Group Funding Opportunity (IRGFO stream 1 and 2) Grant to WJK, Mitacs Globalink Research Internship Program grant to WJK, and Startup funds from HIT Center for Life Science to WJK. This work was also supported by a Brain Pool Program by National Research Foundation in Korea to WJK, Burroughs Wellcome Fund Collaborative Research Travel Grants 1017486 to WJK, NVIDIA Academic Hardware Grant Program to WJK.

## Conflict of interests

The authors have no conflict of interests to disclose.
