# Supplementary material for: Glial subtype-specific modulation of disease pathogenesis in Drosophila models of ALS
Source: Genes Dis. 2025 Apr 8;12(5):101631. doi: 10.1016/j.gendis.2025.101631 (PMC12164006; doi:10.1016/j.gendis.2025.101631)
Supplement: Multimedia component 1 [file mmc1.docx]

**EXPERIMENTAL PROCEDURES**

**Fly Rearing and Strains**

*Drosophila melanogaster* was raised on cornmeal-yeast medium at similar densities to yield adults with similar body sizes. Flies were kept in 12 h light: 12 h dark cycles (LD) at 25°C (zeitgeber time (ZT) 0 is the beginning of the light phase, zeitgeber time (ZT) 12 beginning of the dark phase). Wild-type flies were Canton-S (CS). To reduce the variation from genetic background, all flies were backcrossed for at least 3 generations to CS strain. All mutants and transgenic lines used here have been described previously.

The following lines were obtained from Bloomington Stock Center (#stock number): *nSyb-GAL4* (#51941), *repo-GAL4* (#7415), *nSyb-GAL4* (#51941), *ALG-GAL4* (#45914), *CG-GAL4* (#39944), *EGN-GAL4* (#39157), *EGT-GAL4* (#39908), *PNG-GAL4* (#40436), *SPG-GAL4* (#50472), *SPG-lexA* (#61562), *fru-lexA* (#66698), *UAS-hSOD1-WT* (#64386), *UAS-hSOD1-G85R* (#33608), *UAS-hSOD1-A4V* (#33607), *UAS-Als2-RNAi* (#28533), *UAS-TDP43.YFP* (#79589), *UAS-TBPH-RNAi* (#29517), *UAS-TBPH-RNAi* (#39014), *UAS-TDP43.Q331K* (#79590), *UAS-FIG4-RNAi* (#58063), *UAS-FIG4-WT* (#38291), *UAS-* *ATXN2.32Q* (#79593), *UAS-EWSR1-WT* (#79592), *UAS-GR.PO100* (#58696), *tub-GAL80^ts^* (#7017), *UAS-mCD8GFP* (#5130), *UAS-RedStinger* (#8547), *UAS-mCD8RFP, lexAop-mCD8GFP* (#32229). All MATR3 lines (*UAS-MATR3-WT*, *UAS-MATR3-S85C*, *UAS-MATR3-T622A*, *UAS-MATR3-F115C*) are kindly sent by Dr. Jeehye Park.

**Climbing Assay**

For climbing assay, we modified the conventional RING assay ^1^. In brief, 40-50 aged flies were placed in an empty vial and were tapped to the bottom of the tube. We used 5 days old adults as young flies and 20 days old as old files. After tapping of flies, we recorded 10 seconds of video clip. This experiment was done five times with 5-minute intervals. With recorded video files, we captured the position of flies 10 seconds after tapping the vial. This captured image file was then loaded in ImageJ to perform particle analysis. For quantifying the location of flies inside a vial, we used the "analyze particles" function of ImageJ ^2^. The position of pixels was normalized by height of vial then only the particles above the midline (4 cm) of vial were counted.

**Lifespan Assay and Statistical Analysis**

For lifespan analysis, we employed a standard protocol as we described before ^3^. Briefly, approximately 50 male or female flies of each genotype were collected in vials and raised in typical 12 h light: 12 h dark cycles at either 29°C or 20°C, as indicated for each experiment. Daily mortality counts were recorded for a period of 40 days. Every 3-4 days, surviving flies were transferred to fresh vials and the number of dead flies was recorded. To compare the survival curves of different genotypes, we utilized Kaplan-Meier survival analysis and performed log-rank (Mantel-Cox) tests for statistical significance using GraphPad Prism software. **** = p<0.0001, *** = p < 0.001, ** = p < 0.01, * = p < 0.05, n.s. stands for non-significant differences.

**Adult Leg Dissection and Live Imaging**

Aged flies were dissected under chilled PBS buffer then washed several times with cold PBS. Washed legs and wings then transferred to slide glass then mounted with cover glass filled with 50% glycerol solution. Dissected samples were immediately imaged by Zeiss AxioImager M2. DIC channel was used for taking images of external organs.

**Quantitative Analysis of GFP Fluorescence**

To quantify the GFP and RFP signals in adult fly legs (Fig. 17, Fig. 18, Fig. 19, and Fig. 20), we measured fluorescence intensity using the measure tool of ImageJ (National Institutes of Health, http://rsb.info.nih.gov/ij) as described previously ^4,5^. For quantifying GFP and RFP signals in Fig. 20, we used the "analyze particles" function of ImageJ. Number of particles represents the number of cell nuclei labeled by GAL4 driver. Average size represents the average size of analyzed particles. Percent of area represents percent of area that is covered with particles normalized by total area. Mean intensity represents the mean fluorescence intensity of fluorescent signal thus can be interpreted as the expression level.

**Statistical Analysis**

Statistical analysis of climbing assay was similar with our previous studies ^4,5^. 40-50 adults were used for climbing assay. Statistical comparisons were made between groups that were naively reared or sexually experienced within each experiment. As climbing data of adults showed normal distribution (Kolmogorov-Smirnov tests, p > 0.05), we used two-sided Student's t tests. Each figure shows the mean ± standard error (s.e.m) (**** = p<0.0001, *** = p < 0.001, ** = p < 0.01, * = p < 0.05). All analysis was done in GraphPad (Prism). Individual tests and significance are detailed in figure legends.

**Single-nucleus RNA-sequencing analyses—Data and code availability**

snRNAseq dataset analyzed in this paper is published in ^6^ and available at the Nextflow pipelines (VSN, https://github.com/vib-singlecell-nf), the availability of raw and processed datasets for users to explore, and the development of a crowd-annotation platform with voting, comments, and references through SCope (https://flycellatlas.org/scope), linked to an online analysis platform in ASAP (<https://asap.epfl.ch/fca>).

**REFERENCES**

1. Gargano JW, Martin I, Bhandari P, Grotewiel MS. Rapid iterative negative geotaxis (RING): a new method for assessing age-related locomotor decline in Drosophila. *Exp Gerontol*. 2005;40(5):386-395. doi:10.1016/j.exger.2005.02.005

2. Grishagin IV. Automatic cell counting with ImageJ. *Anal Biochem*. 2015;473:63-65. doi:10.1016/j.ab.2014.12.007

3. Piper MDW, Partridge L. Protocols to Study Aging in Drosophila. In: Springer New York; 2016:291-302. doi:10.1007/978-1-4939-6371-3_18

4. Miao H, Wei Y, Lee SG, Wu Z, Kaur J, Kim WJ. Glia‐specific expression of neuropeptide receptor Lgr4 regulates development and adult physiology in Drosophila. *J Neurosci Res*. 2024;102(1). doi:10.1002/jnr.25271

5. Zhang X, Sun D, Wong K, Salkini A, Najafi H, Kim WJ. The astrocyte-enriched gene deathstar plays a crucial role in the development, locomotion, and lifespan of D. melanogaster. *Fly*. 2024;18(1):2368336. doi:10.1080/19336934.2024.2368336

6. Li H, Janssens J, Waegeneer MD, et al. Fly Cell Atlas: A single-nucleus transcriptomic atlas of the adult fruit fly. *Science*. 2022;375(6584):eabk2432. doi:10.1126/science.abk2432

**FIGURE LEGENDS**

**Fig. SD1.** Climbing test and lifespan of flies expressing SOD1-WT by different neuronal and glial drivers. (A-D) Climbing ability of pan-neuronal and glial SOD1-WT expressing flies, 5-day-old and 20-day-old females (A-B) and males (C-D). (E-F) Lifespan of neuronal and glial SOD1-WT expressing flies, females (E) and males (F). (G) t-distributed stochastic neighbor embedding (t-SNE) representation of scRNA-seq datasets. t-SNE plot showing *Sod1* (red), PNG (blue), SPG (green) in whole body of *Drosophila*. (H-I) Climbing test of flies expressing SOD1-WT by subtype glial drivers, 5-day-old (H) and 20-day-old (I) females. All experiments were performed five times after sufficient recovering period. Genotypes are labeled below the graph. Rearing temperature, age, number, and sex of animals are labeled within the graph. Box represents min to max that show all points of data. The median value and standard error are labeled within the box-and-whisker plot (black lines). Mean value is labeled as cross mark (+) within box. Asterisks represent significant differences revealed by unpaired Student's t test (* p<0.05, ** p<0.01, *** p<0.001, **** p<0.0001). n.s. represents non-significant differences revealed by unpaired Student's t test, and was not marked out in climbing test figures of subtype glia cells. The same notations of climbing assay for statistical analysis are used in other figures. See **EXPERIMENTAL PROCEDURES** and previous report for detailed quantification methods.

**Fig. SD2.** Climbing test and lifespan of flies expressing SOD1-G85R by different neuronal and glial drivers. (A-D) Climbing ability of pan-neuronal and glial SOD1-G85R expressing flies, 5-day-old and 20-day-old females (A-B) and males (C-D). (E-F) Lifespan of neuronal and glial SOD1-G85R expressing flies, females (E) and males (F). (G-H) Climbing test of flies expressing SOD1-G85R by subtype glial drivers, 5-day-old (G) and 20-day-old (H) females.

**Fig. SD3.** Climbing test and lifespan of flies expressing SOD1-A4V by different neuronal and glial drivers. (A-D) Climbing ability of pan-neuronal and glial SOD1-A4V expressing flies, 5-day-old and 20-day-old females (A-B) and males (C-D). (E-F) Lifespan of neuronal and glial SOD1-A4V expressing flies, females (E) and males (F). (G-H) Climbing test of flies expressing SOD1-A4V by subtype glial drivers, 5-day-old (G) and 20-day-old (H) females.

**Fig. SD4.** Climbing test and lifespan of flies knocking down Als2 by different neuronal and glial drivers. (A-D) Climbing ability of pan-neuronal and glial Als2 knocking down flies, 5-day-old and 20-day-old females (A-B) and males (C-D). (E-F) Lifespan of neuronal and glial Als2-RNAi flies, females (E) and males (F). (G) t-distributed stochastic neighbor embedding (t-SNE) representation of scRNA-seq datasets. t-SNE plot showing *Als2* (red), PNG (blue), SPG (green) in whole body of *Drosophila*. (H-I) Climbing test of flies knocking down Als2 by subtype glial drivers, 5-day-old (H) and 20-day-old (I) females.

**Fig. SD5.** Climbing test and lifespan of flies expressing TDP43.YFP by different neuronal and glial drivers. (A-D) Climbing ability of pan-neuronal and glial TDP43.YFP expressing flies, 5-day-old and 20-day-old females (A-B) and males (C-D). (E-F) Lifespan of neuronal and glial TDP43.YFP expressing flies, females (E) and males (F). (G) t-distributed stochastic neighbor embedding (t-SNE) representation of scRNA-seq datasets. t-SNE plot showing *TBPH* (red), PNG (blue), SPG (green) in whole body of *Drosophila*. (H-I) Climbing test of flies expressing TDP43.YFP by subtype glial drivers, 5-day-old (H) and 20-day-old (I) females.

**Fig. SD6.** Climbing test and lifespan of flies knocking down TBPH^29517^ by different neuronal and glial drivers. (A-D) Climbing ability of pan-neuronal and glial TBPH^29517^ knocking down flies, 5-day-old and 20-day-old females (A-B) and males (C-D). (E-F) Lifespan of neuronal and glial TBPH-RNAi^29517^ flies, females (E) and males (F). (G) t-distributed stochastic neighbor embedding (t-SNE) representation of scRNA-seq datasets. t-SNE plot showing *TBPH* (red), PNG (blue), SPG (green) in whole body of *Drosophila*. (H-I) Climbing test of flies knocking down TBPH^29517^ by subtype glial drivers, 5-day-old (H) and 20-day-old (I) females.

**Fig. SD7.** Climbing test and lifespan of flies knocking down TBPH^39014^ by different neuronal and glial drivers. (A-D) Climbing ability of pan-neuronal and glial TBPH^39014^ knocking down flies, 5-day-old and 20-day-old females (A-B) and males (C-D). (E-F) Lifespan of neuronal and glial TBPH-RNAi^39014^ flies, females (E) and males (F). (G) t-distributed stochastic neighbor embedding (t-SNE) representation of scRNA-seq datasets. t-SNE plot showing *TBPH* (red), PNG (blue), SPG (green) in whole body of *Drosophila*. (H-K) Climbing test of flies knocking down TBPH^39014^ by subtype glial drivers, 5-day-old and 20-day-old females (H-I) and males (J-K). (L-N) Climbing test of flies knocking down TBPH^39014^ by subtype glial drivers, ALG and CG drivers (L), EGN and EGT drivers (M), PNG and SPG drivers (N).

**Fig. SD8.** Climbing test and lifespan of flies expressing TDP43-Q331K by different neuronal and glial drivers. (A-D) Climbing ability of pan-neuronal and glial TDP43-Q331K expressing flies, 5-day-old and 20-day-old females (A-B) and males (C-D). (E-F) Lifespan of neuronal and glial TDP43-Q331K expressing flies, females (E) and males (F). (G) t-distributed stochastic neighbor embedding (t-SNE) representation of scRNA-seq datasets. t-SNE plot showing *TBPH* (red), PNG (blue), SPG (green) in whole body of *Drosophila*. (H-K) Climbing test of flies expressing TDP43-Q331K by subtype glial drivers, 5-day-old and 20-day-old females (H-I) and males (J-K). (L-N) Climbing test of flies expressing TDP43-Q331K by subtype glial drivers, ALG and CG drivers (L), EGN and EGT drivers (M), PNG and SPG drivers (N).

**Fig. SD9.** Climbing test and lifespan of flies knocking down FIG4 by different neuronal and glial drivers. (A-D) Climbing ability of pan-neuronal and glial FIG4 knocking down flies, 5-day-old and 20-day-old females (A-B) and males (C-D). (E-F) Lifespan of neuronal and glial FIG4-RNAi flies, females (E) and males (F). (G) t-distributed stochastic neighbor embedding (t-SNE) representation of scRNA-seq datasets. t-SNE plot showing *FIG4* (red), PNG (blue), SPG (green) in whole body of *Drosophila*. (H-K) Climbing test of flies knocking down FIG4 by subtype glial drivers, 5-day-old and 20-day-old females (H-I) and males (J-K). (L-N) Climbing test of flies knocking down FIG4 by subtype glial drivers, ALG and CG drivers (L), EGN and EGT drivers (M), PNG and SPG drivers (N).

**Fig. SD10.** Climbing test and lifespan of flies expressing FIG-RNAi by different neuronal and glial drivers. (A-D) Climbing ability of pan-neuronal and glial FIG-RNAi expressing flies, 5-day-old and 20-day-old females (A-B) and males (C-D). (E-F) Lifespan of neuronal and glial FIG-RNAi expressing flies, females (E) and males (F). (G) t-distributed stochastic neighbor embedding (t-SNE) representation of scRNA-seq datasets. t-SNE plot showing *FIG4* (red), PNG (blue), SPG (green) in whole body of *Drosophila*. (H-I) Climbing test of flies expressing FIG-RNAi by subtype glial drivers, 5-day-old (H) and 20-day-old (I) females.

**Fig. SD11.** Climbing test and lifespan of flies expressing ATXN2.32Q by different neuronal and glial drivers. (A-D) Climbing ability of pan-neuronal and glial ATXN2.32Q expressing flies, 5-day-old and 20-day-old females (A-B) and males (C-D). (E-F) Lifespan of neuronal and glial ATXN2.32Q expressing flies, females (E) and males (F). (G) t-distributed stochastic neighbor embedding (t-SNE) representation of scRNA-seq datasets. t-SNE plot showing *Atx2* (red), PNG (blue), SPG (green) in whole body of *Drosophila*. (H-I) Climbing test of flies expressing ATXN2.32Q by subtype glial drivers, 5-day-old (H) and 20-day-old (I) females.

**Fig. SD12.** Climbing test and lifespan of flies expressing EWSR1-WT by different neuronal and glial drivers. (A-D) Climbing ability of pan-neuronal and glial EWSR1-WT expressing flies, 5-day-old and 20-day-old females (A-B) and males (C-D). (E-F) Lifespan of neuronal and glial EWSR1-WT expressing flies, females (E) and males (F). (G) t-distributed stochastic neighbor embedding (t-SNE) representation of scRNA-seq datasets. t-SNE plot showing *caz* (red), PNG (blue), SPG (green) in whole body of *Drosophila*. (H-I) Climbing test of flies expressing EWSR1-WT by subtype glial drivers, 5-day-old (H) and 20-day-old (I) females.

**Fig. SD13.** Lifespan and climbing test of flies expressing different MATR3 genes by neuronal and glial drivers. (A-B) Lifespan of pan-neuronal and glial MATR3-WT expressing flies, males (A) and females (B). (C-F) Climbing ability of neuronal and glial MATR3-S85C expressing flies, 5-day-old and 20-day-old females (C-D) and males (E-F). (H-K) Climbing ability of neuronal and glial MATR3-T622A expressing flies, 5-day-old and 20-day-old females (H-I) and males (J-K). (G-L) Lifespan of pan-neuronal and glial MATR3-S85C expressing male flies (G), and MATR3-T622A expressing male flies (L).

**Fig. SD14.** Climbing test and lifespan of flies expressing MATR3-F115C by neuronal and glial drivers. (A-B) Climbing ability of pan-neuronal and glial MATR3-F115C expressing flies, 5-day-old (A) and 20-day-old (B) females. (C) Lifespan of pan-neuronal and glial MATR3-F115C expressing male flies. (D) Lifespan of pan-neuronal and glial MATR3-F115C expressing female flies.

**Fig. SD15.** Surface glia and neurons in adult foreleg. (A-C) Male foreleg expressing (A) *UAS-mCD8RFP* together with *nSyb-GAL4* and (B) *lexAop-mCD8GFP* together with *SPG-lexA* were imaged live under fluorescent microscope and (C) merged. (D-F) Female foreleg expressing (D) *UAS-mCD8RFP* together with *nSyb-GAL4* and (E) *lexAop-mCD8GFP* together with *SPG-lexA* were imaged live under fluorescent microscope and (F) merged. Scale bars represent 50 mm.

**Fig. SD16.** Surface glia and neurons in adult midleg and hindleg. (A-C) Male midleg expressing (A) *UAS-mCD8RFP* together with *nSyb-GAL4* and (B) *lexAop-mCD8GFP* together with *SPG-lexA* were imaged live under fluorescent microscope and (C) merged. (D-F) Female midleg expressing (D) *UAS-mCD8RFP* together with *nSyb-GAL4* and (E) *lexAop-mCD8GFP* together with *SPG-lexA* were imaged live under fluorescent microscope and (F) merged. (G-I) Male hindleg expressing (G) *UAS-mCD8RFP* together with *nSyb-GAL4* and (H) *lexAop-mCD8GFP* together with *SPG-lexA* were imaged live under fluorescent microscope and (I) merged. (J-L) Female hindleg expressing (J) *UAS-mCD8RFP* together with *nSyb-GAL4* and (K) *lexAop-mCD8GFP* together with *SPG-lexA* were imaged live under fluorescent microscope and (L) merged. Scale bars represent 50 mm.

**Fig. SD17.** Proportion of surface glia and neurons in adult foreleg. (A) The distribution of male foreleg expressing *UAS-mCD8RFP* together with *nSyb-GAL4* and *lexAop-mCD8GFP* together with *SPG-lexA* and merged. (B) The distribution of female foreleg expressing *UAS-mCD8RFP* together with *nSyb-GAL4* and *lexAop-mCD8GFP* together with *SPG-lexA* and merged. Scale bars represent 50 mm. (C-D) Percent area of SPG (C) and neuron (D) in adult foreleg between male and female. Right Y axis represents estimation plot and the black whiskers span the 95% CIs.

**Fig. SD18.** Proportion of surface glia and neurons in adult midleg. (A) The distribution of male midleg expressing *UAS-mCD8RFP* together with *nSyb-GAL4* and *lexAop-mCD8GFP* together with *SPG-lexA* and merged. (B) The distribution of female midleg expressing *UAS-mCD8RFP* together with *nSyb-GAL4* and *lexAop-mCD8GFP* together with *SPG-lexA* and merged. Scale bars represent 50 mm. (C-D) Percent area of SPG (C) and neuron (D) in adult midleg between male and female. Right Y axis represents estimation plot and the black whiskers span the 95% CIs.

**Fig. SD19.** Proportion of surface glia and neurons in adult hindleg. (A) The distribution of male hindleg expressing *UAS-mCD8RFP* together with *nSyb-GAL4* and *lexAop-mCD8GFP* together with *SPG-lexA* and merged. (B) The distribution of female hindleg expressing *UAS-mCD8RFP* together with *nSyb-GAL4* and *lexAop-mCD8GFP* together with *SPG-lexA* and merged. Scale bars represent 50 mm. (C-D) Percent area of SPG (C) and neuron (D) in adult hindleg between male and female. Right Y axis represents estimation plot and the black whiskers span the 95% CIs.

**Fig. SD20.** Neurons and glial cells in adult foreleg of (A-C) males and (E-G) females. (A-B) Male forelegs or (C-D) wings expressing (A and C) UAS-mCD8RFP together with repo-GAL4 or (B and D) UAS-mCD8GFP together with nSyb-GAL4 were imaged live under fluorescent microscope. (E-F) Female forelegs or (G-H) wings expressing (E and G) UAS-mCD8RFP together with repo-GAL4 or (F and H) UAS-mCD8GFP together with nSyb-GAL4 were imaged live under fluorescent microscope. Scale bars represent 50 mm.

**Table S1.** Summary for Lifespan and Climbing assay phenotypes. The first row of each climbing data is for flies aged 5 days and second for 20 days. “+” : Positive effects; “N”: No effects; “-” : Negative effects; “N/A”: No clear information available.
