# Supplementary figures and images for: Glial subtype-specific modulation of disease pathogenesis in Drosophila models of ALS
Source: Genes Dis. 2025 Apr 8;12(5):101631. doi: 10.1016/j.gendis.2025.101631 (PMC12164006; doi:10.1016/j.gendis.2025.101631)

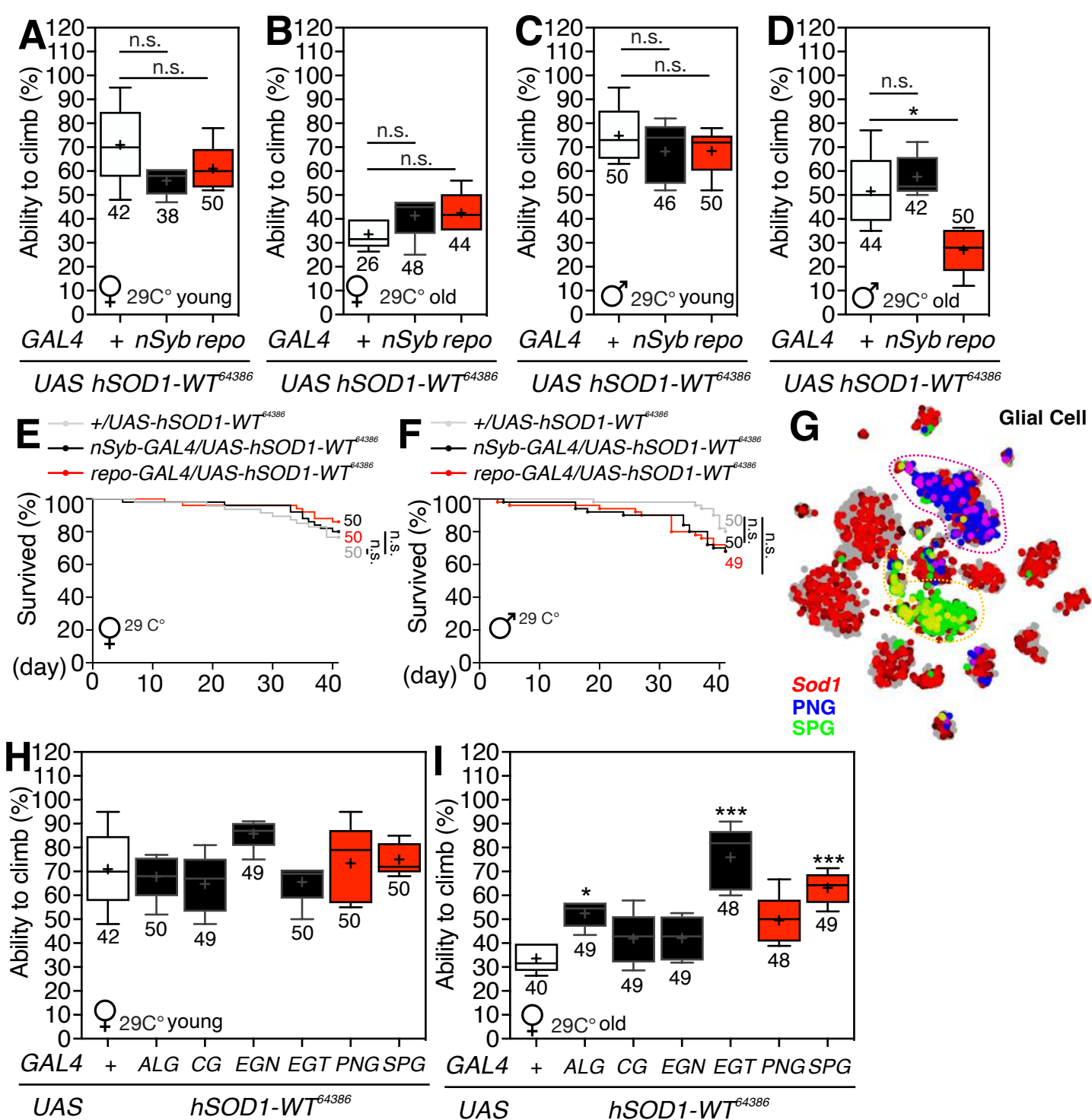

Supplement: Multimedia component 2 [file mmc2.pdf]

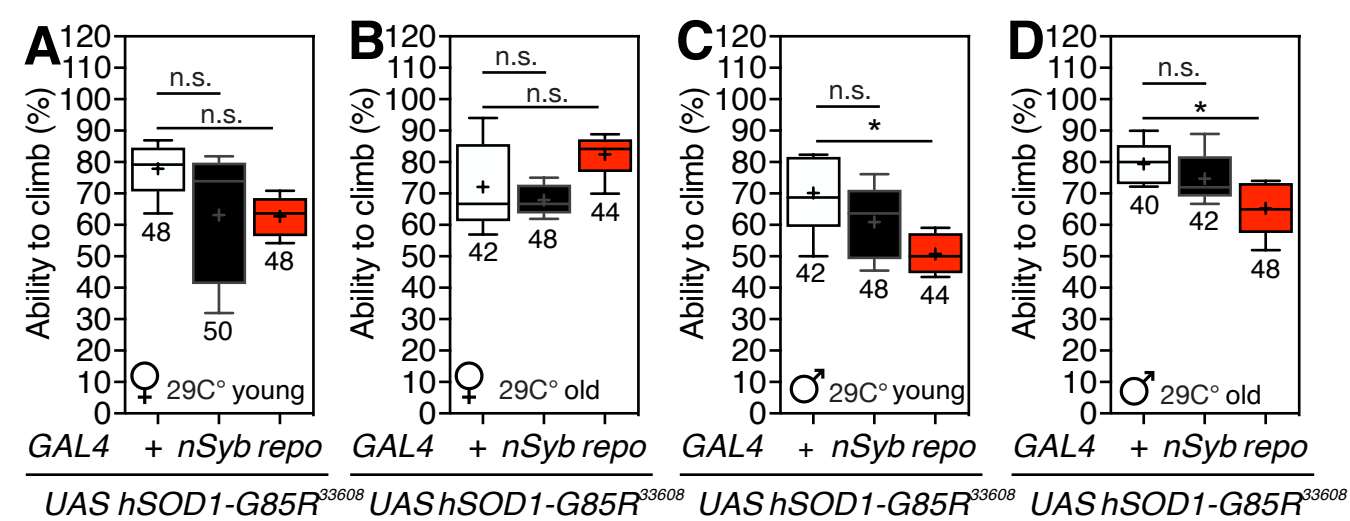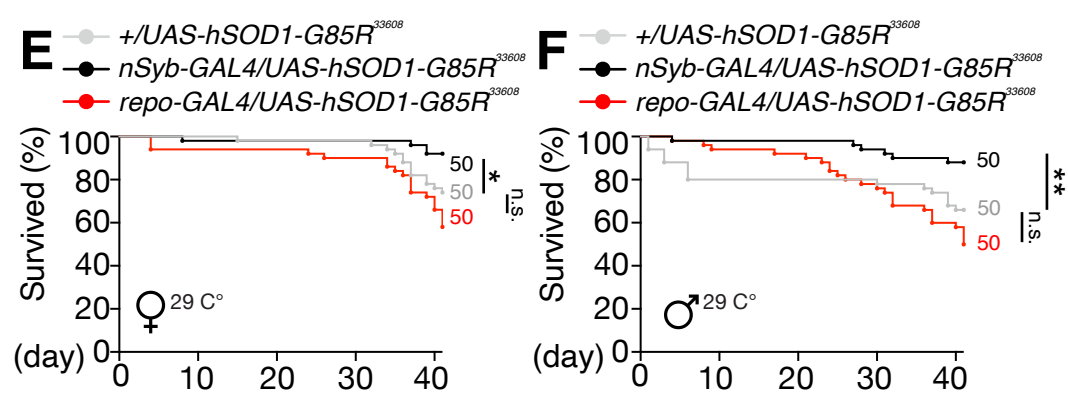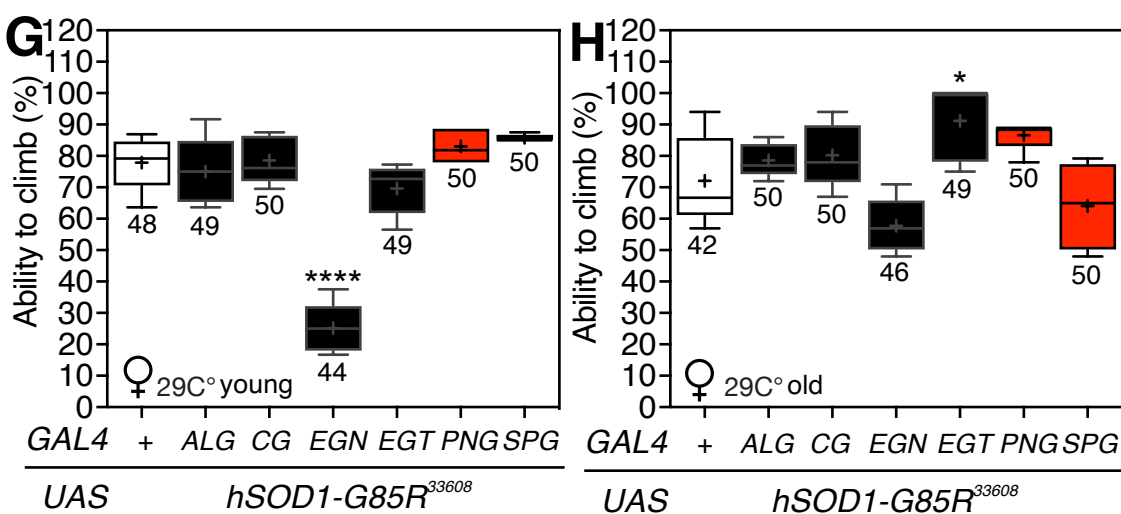

Supplement: Multimedia component 3 [file mmc3.pdf]

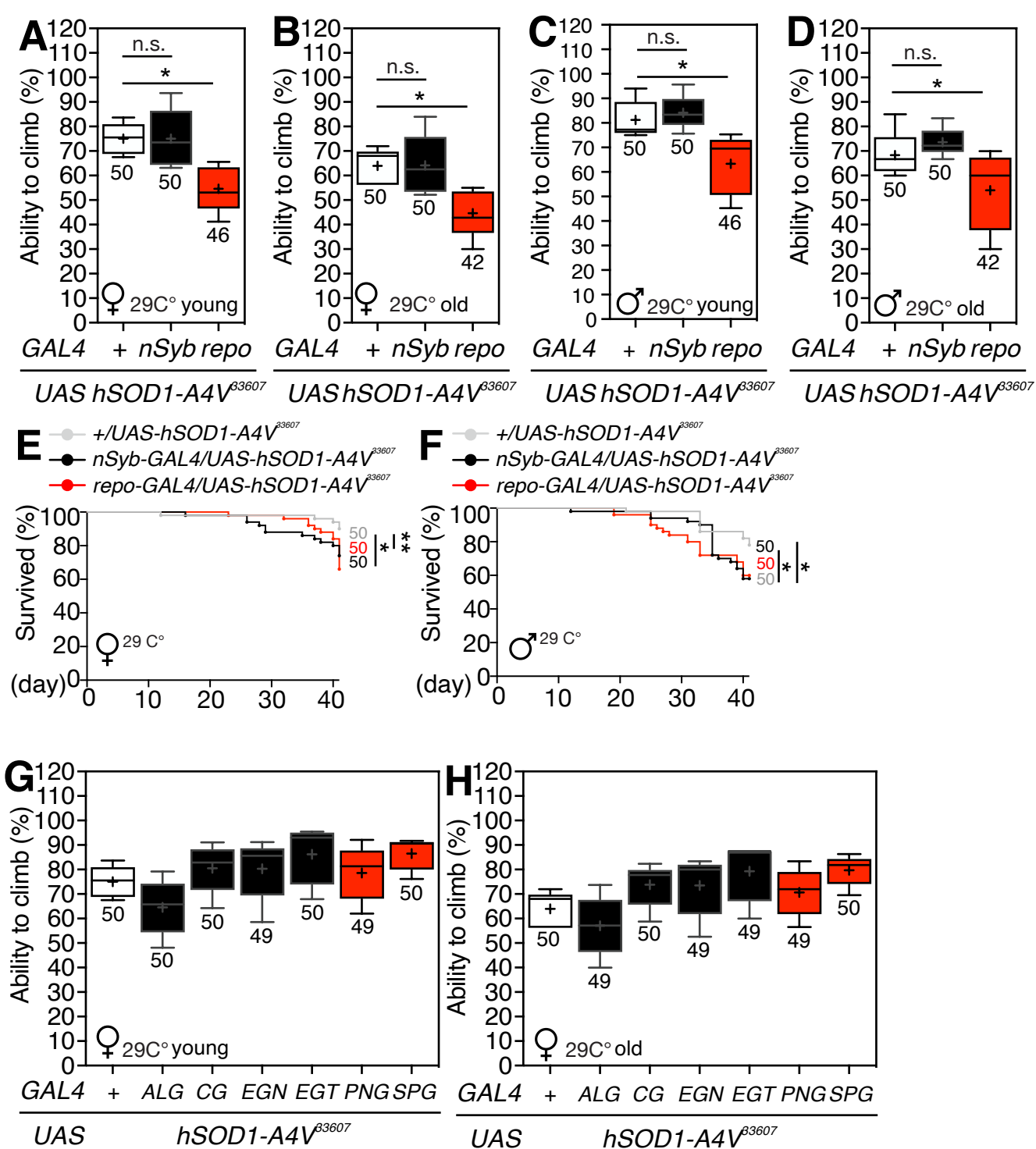

Supplement: Multimedia component 4 [file mmc4.pdf]

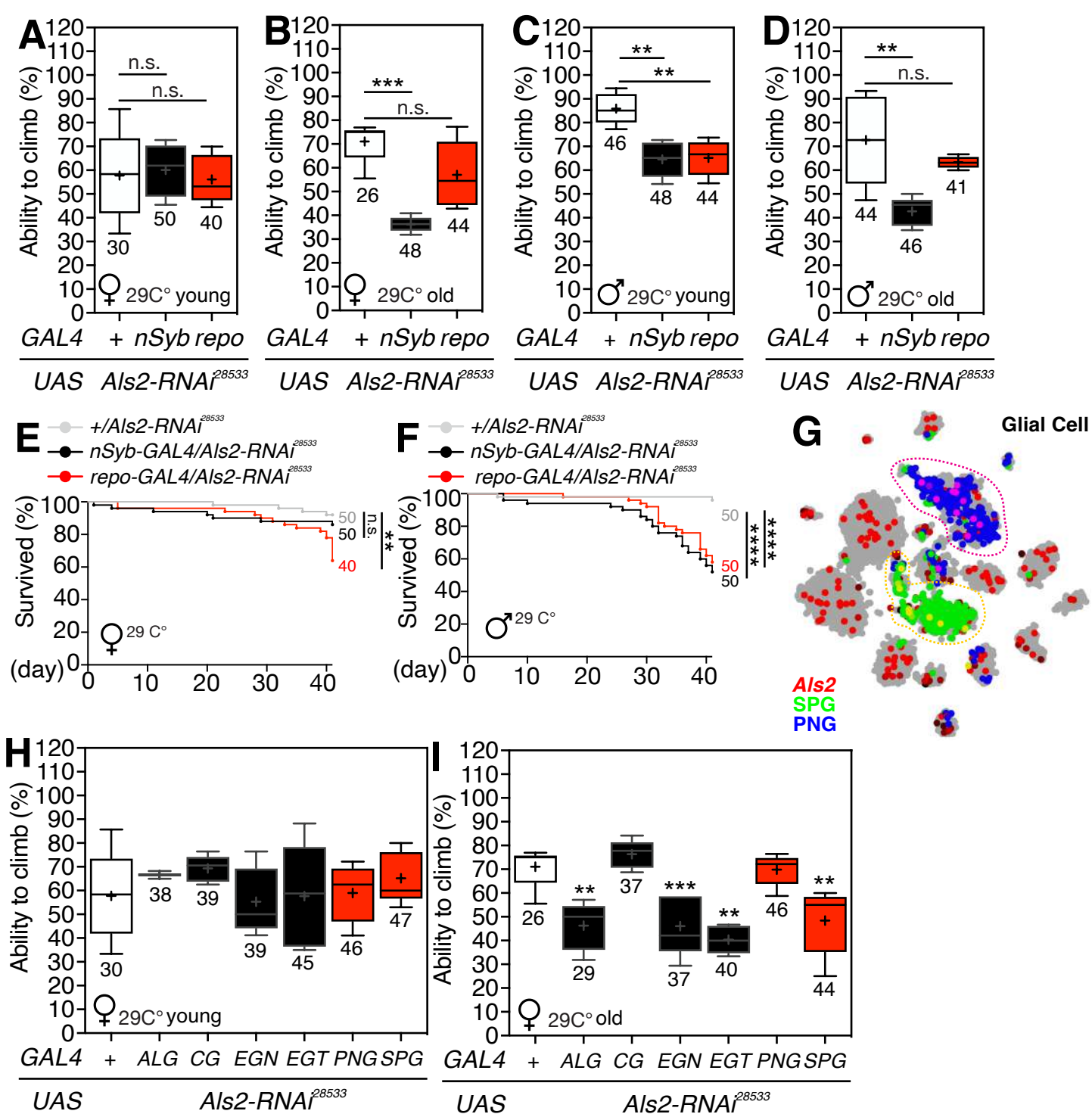

Supplement: Multimedia component 5 [file mmc5.pdf]

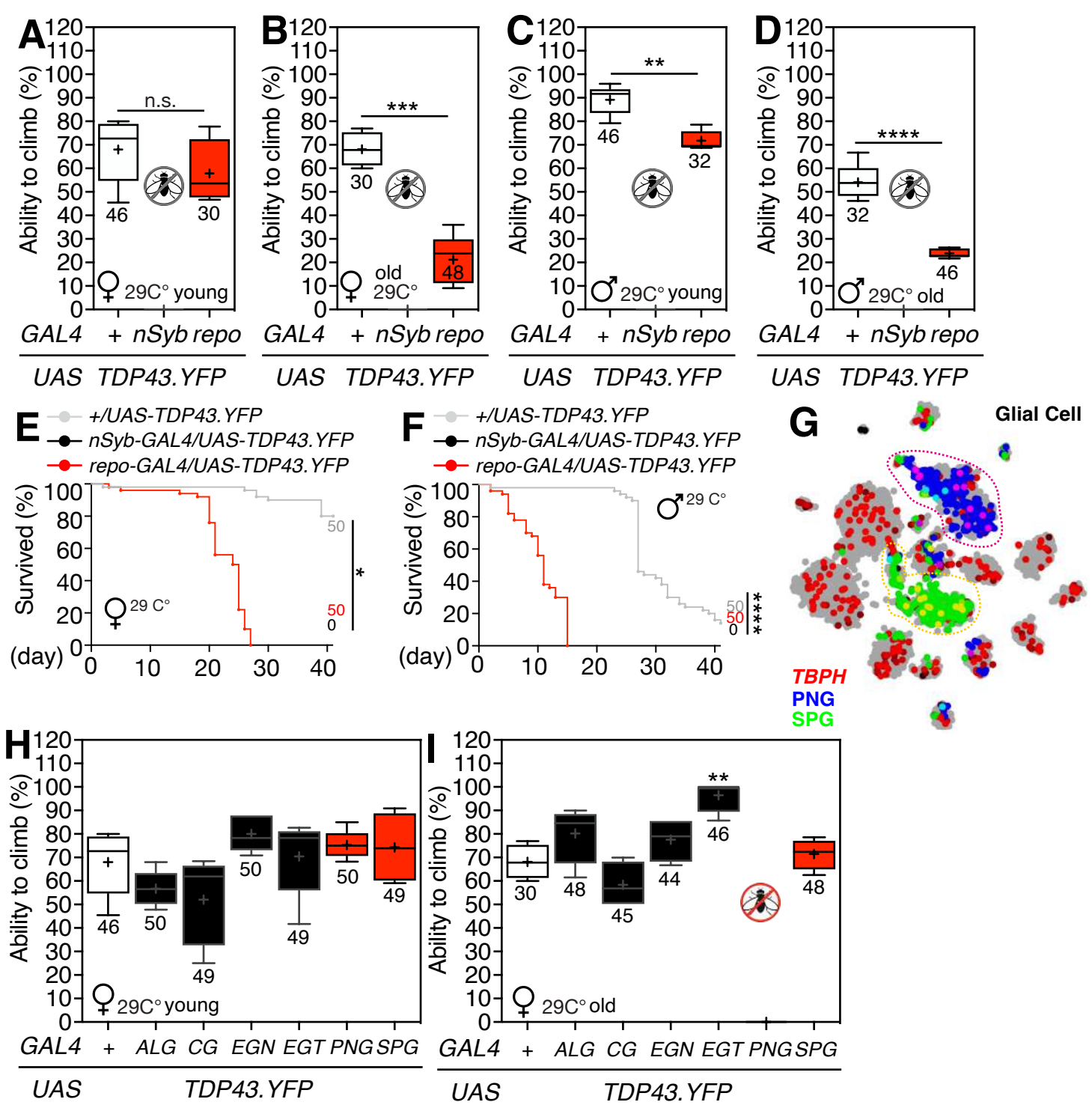

Supplement: Multimedia component 6 [file mmc6.pdf]

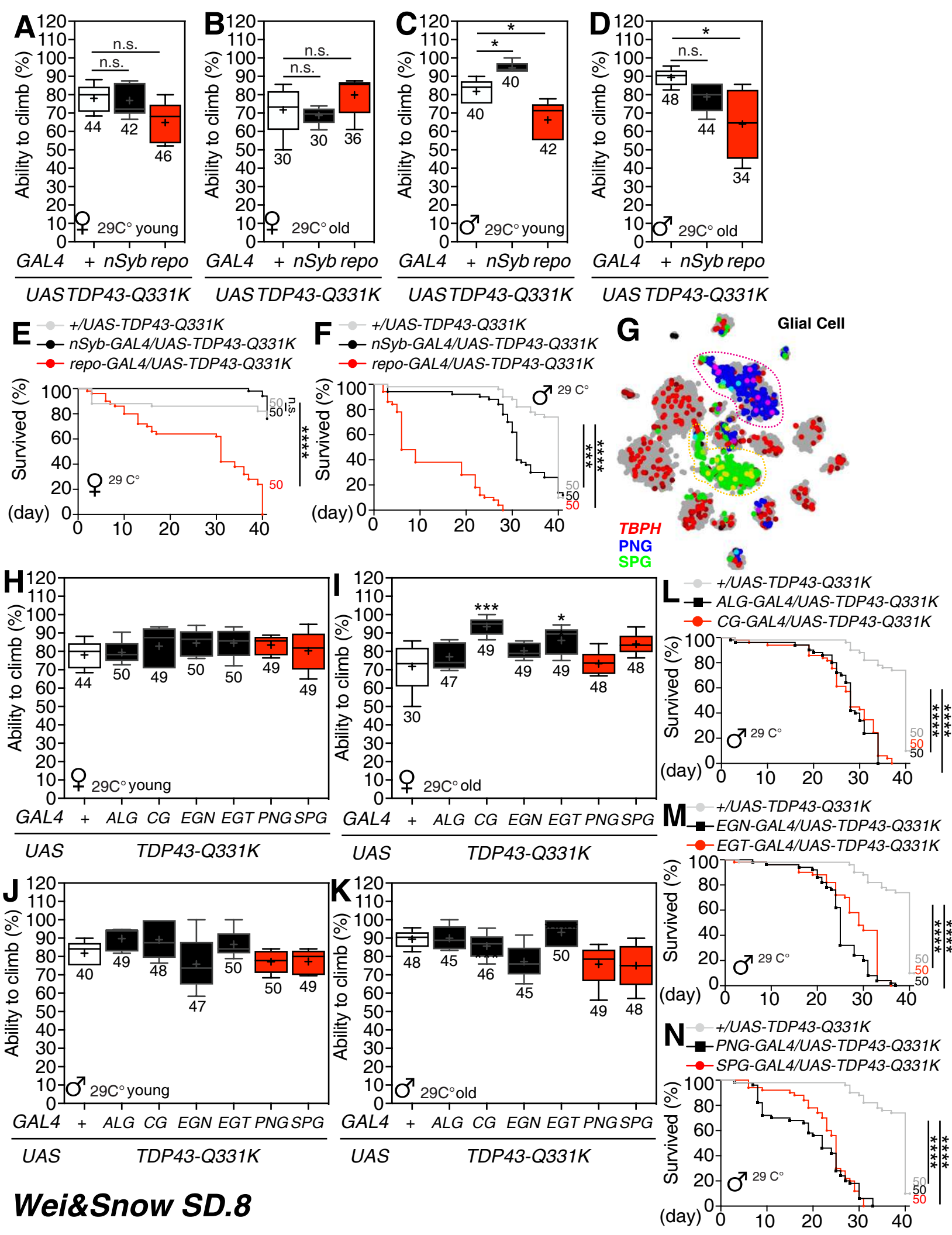

Supplement: Multimedia component 9 [file mmc9.pdf]

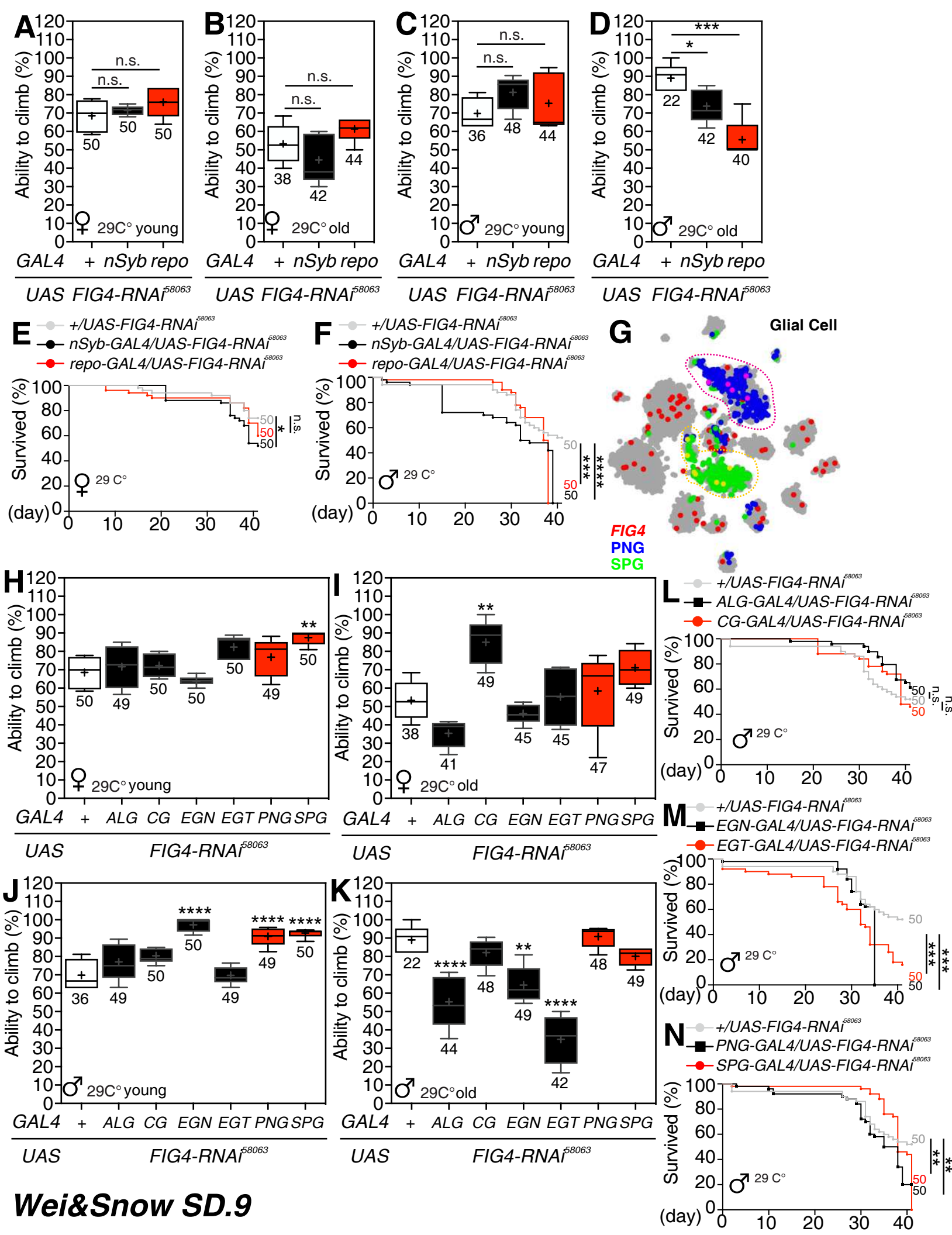

Supplement: Multimedia component 10 [file mmc10.pdf]

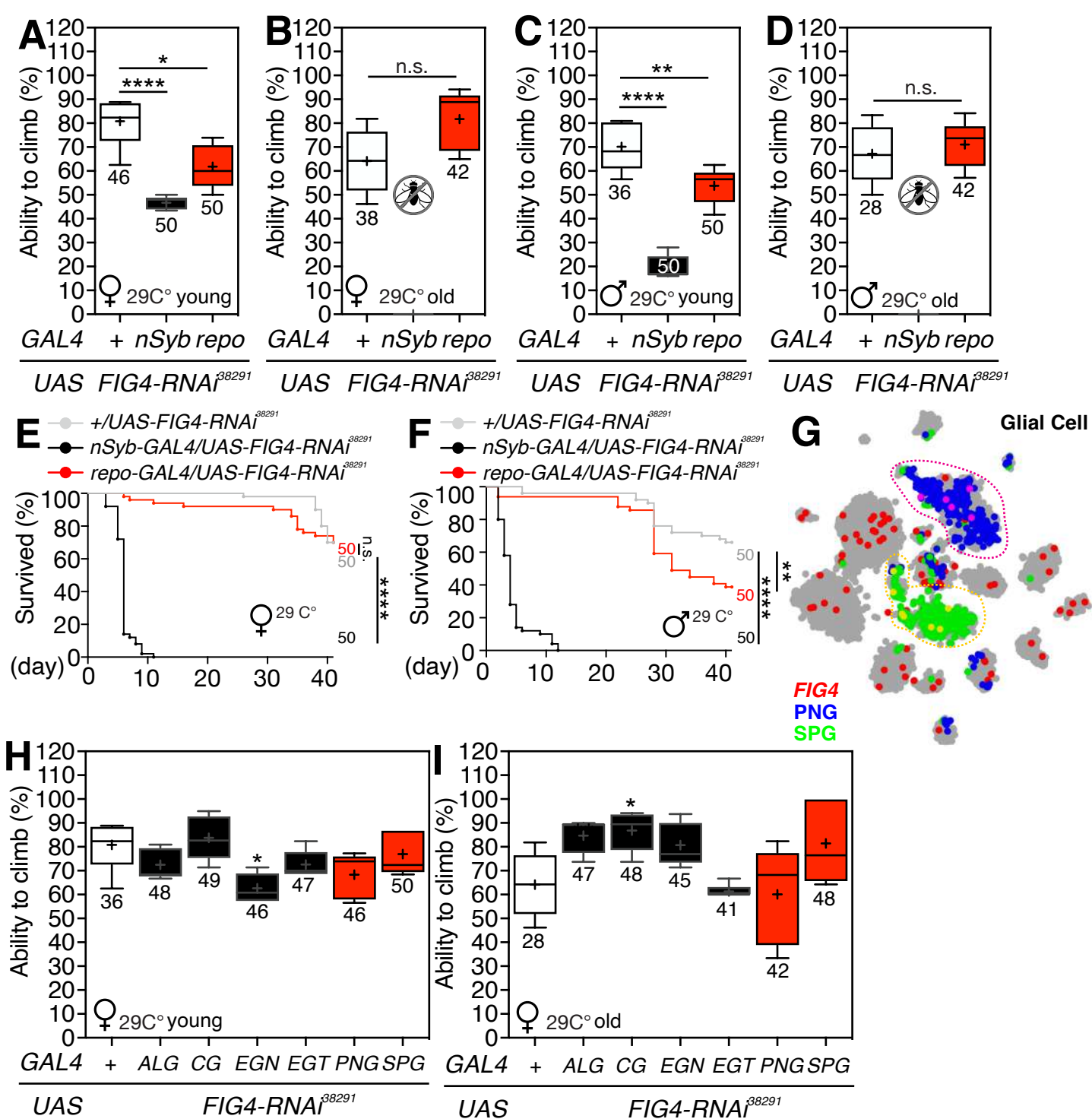

Supplement: Multimedia component 11 [file mmc11.pdf]

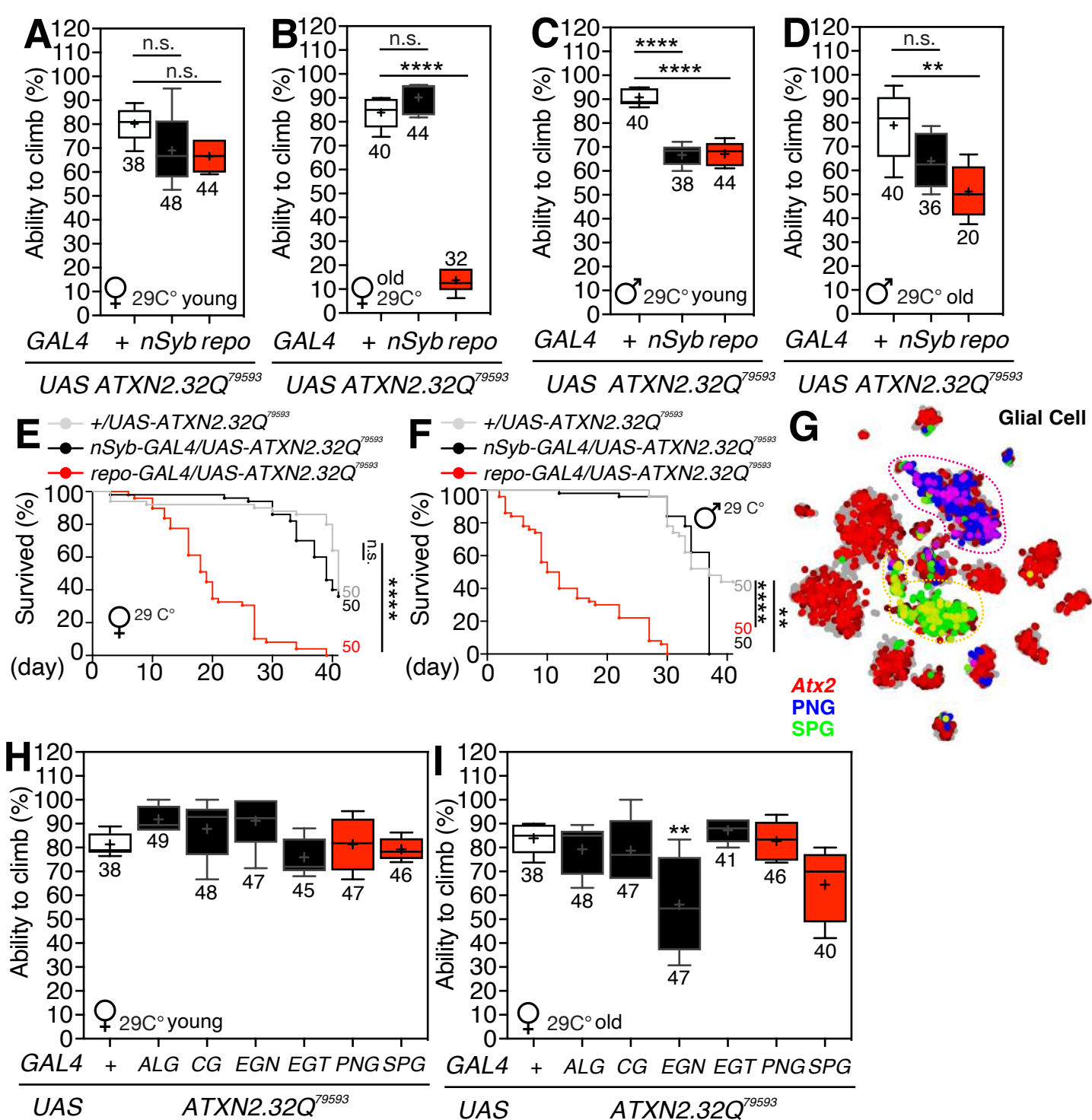

Supplement: Multimedia component 12 [file mmc12.pdf]

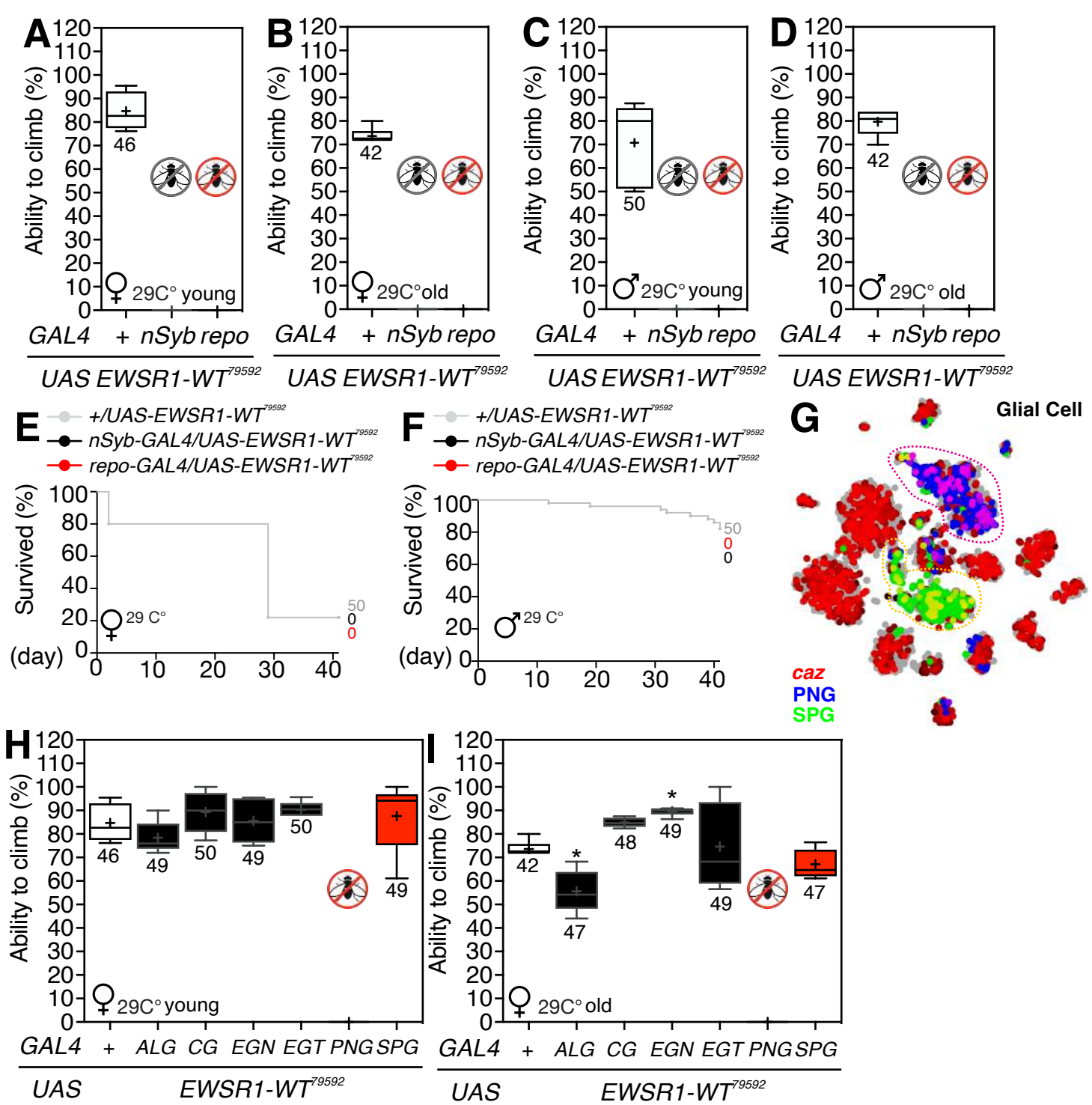

Supplement: Multimedia component 13 [file mmc13.pdf]

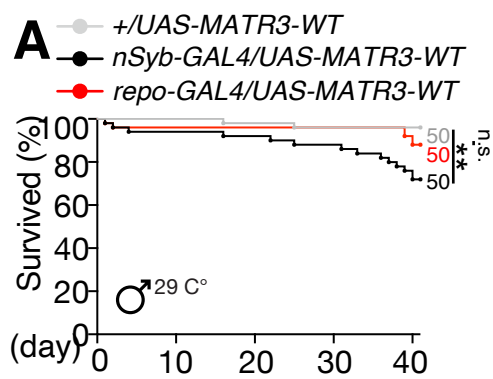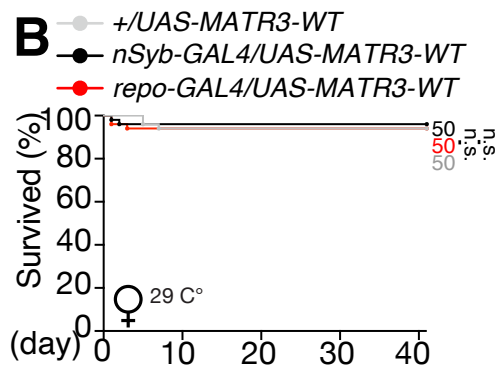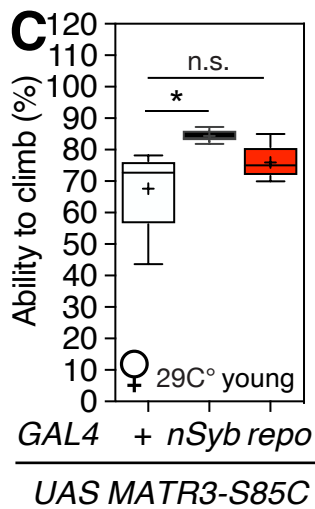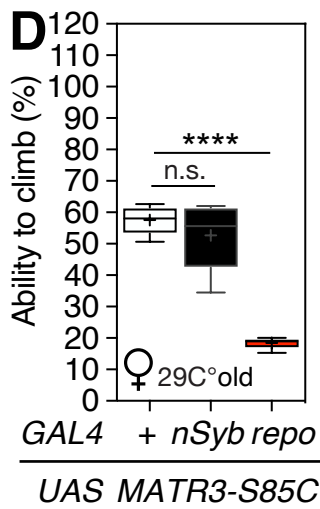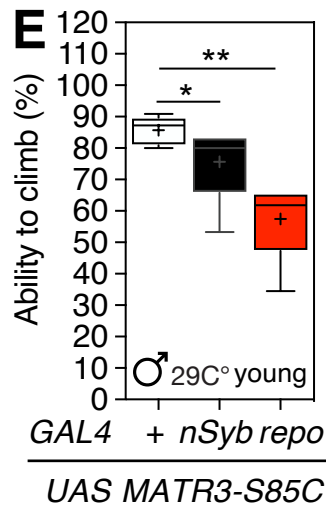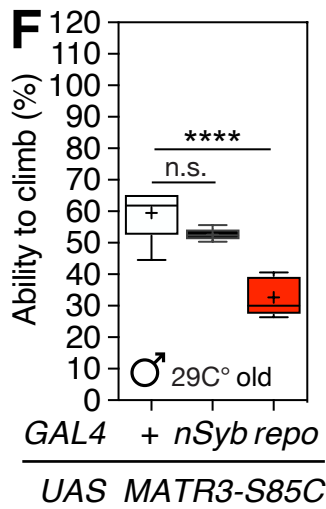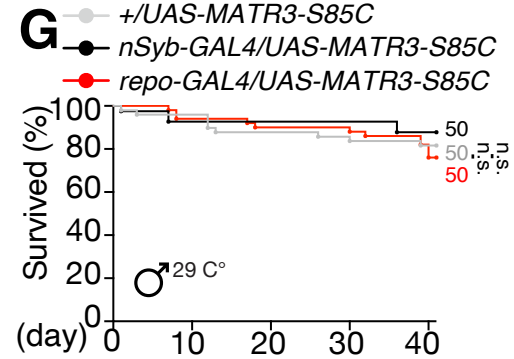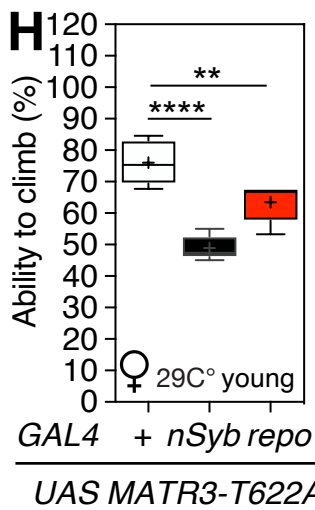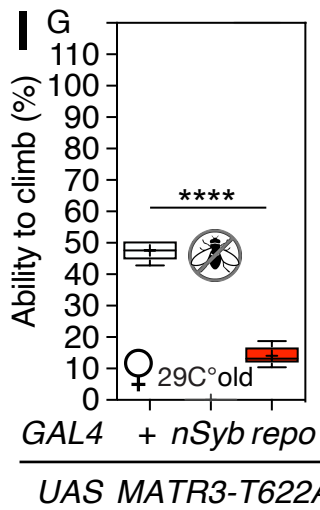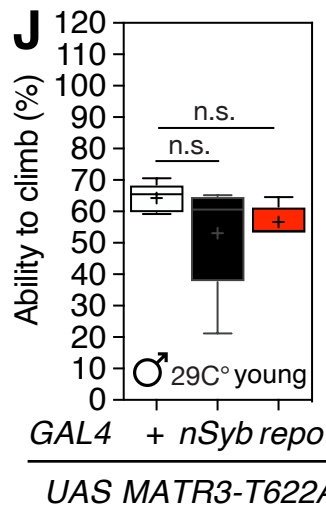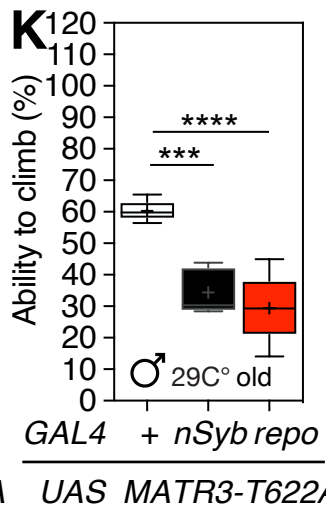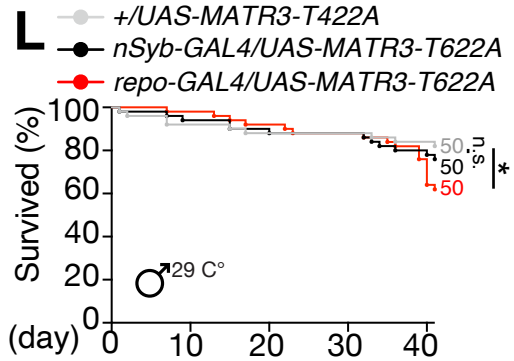

Supplement: Multimedia component 14 [file mmc14.pdf]

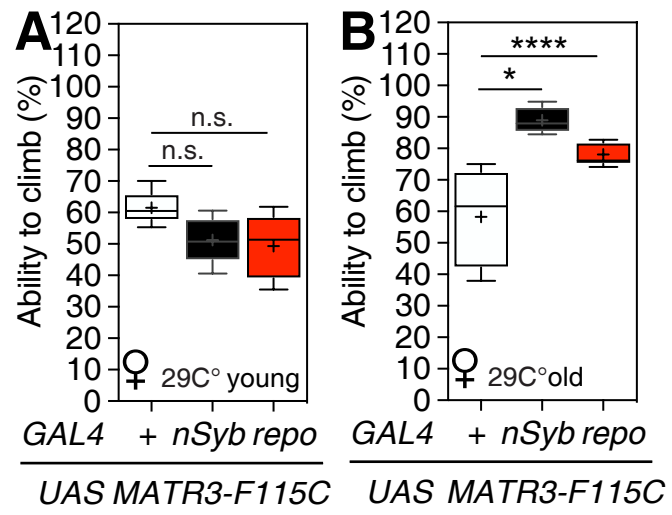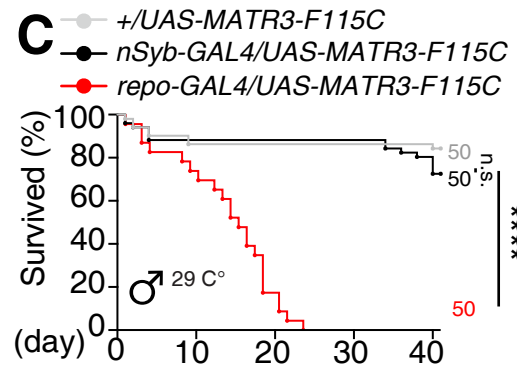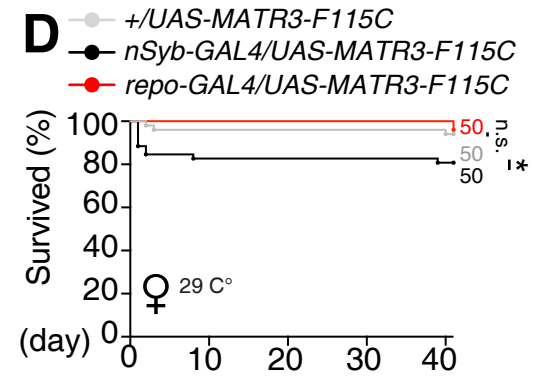

Supplement: Multimedia component 15 [file mmc15.pdf]

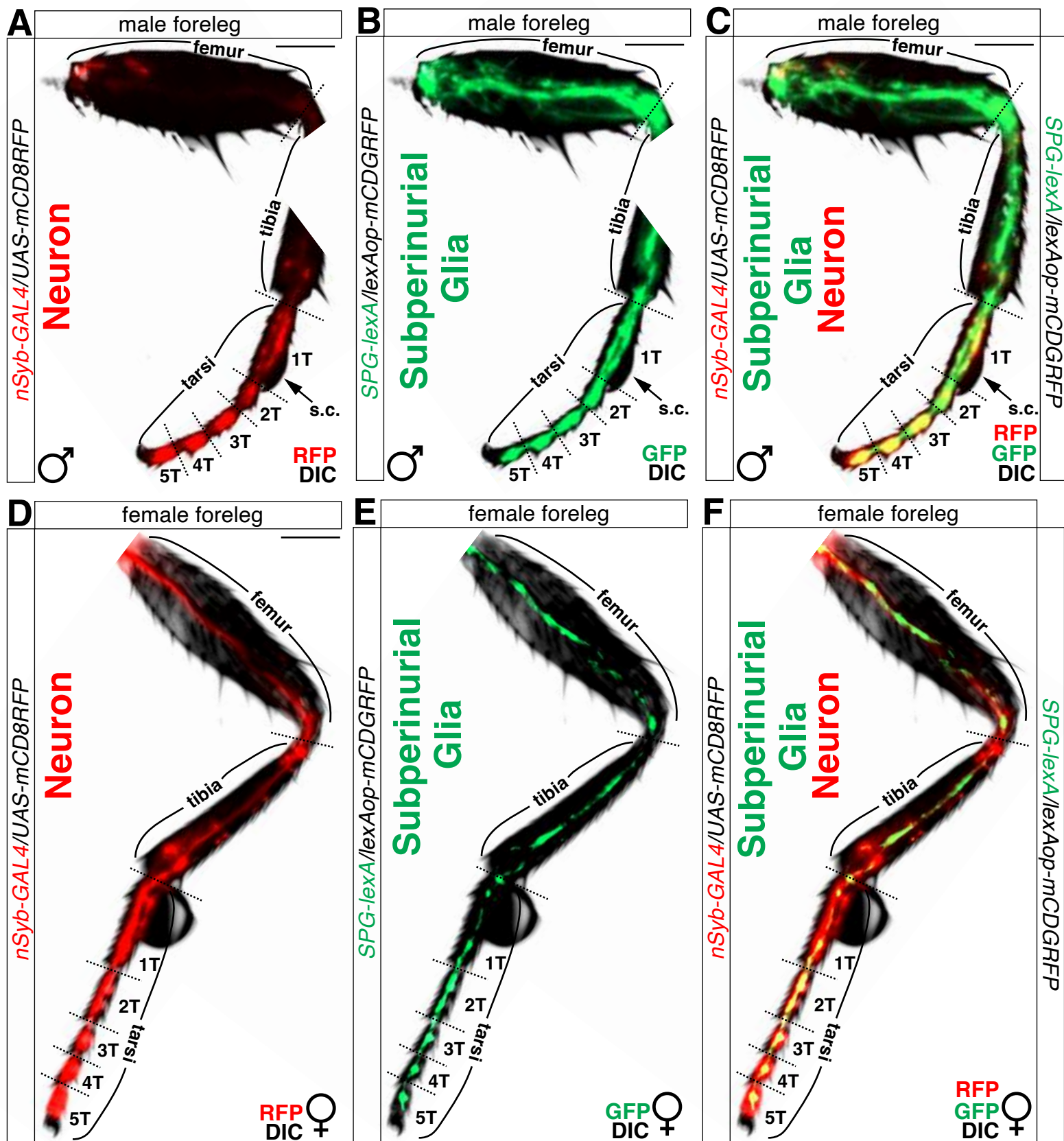

*Snow., SD.15*

Supplement: Multimedia component 16 [file mmc16.pdf]

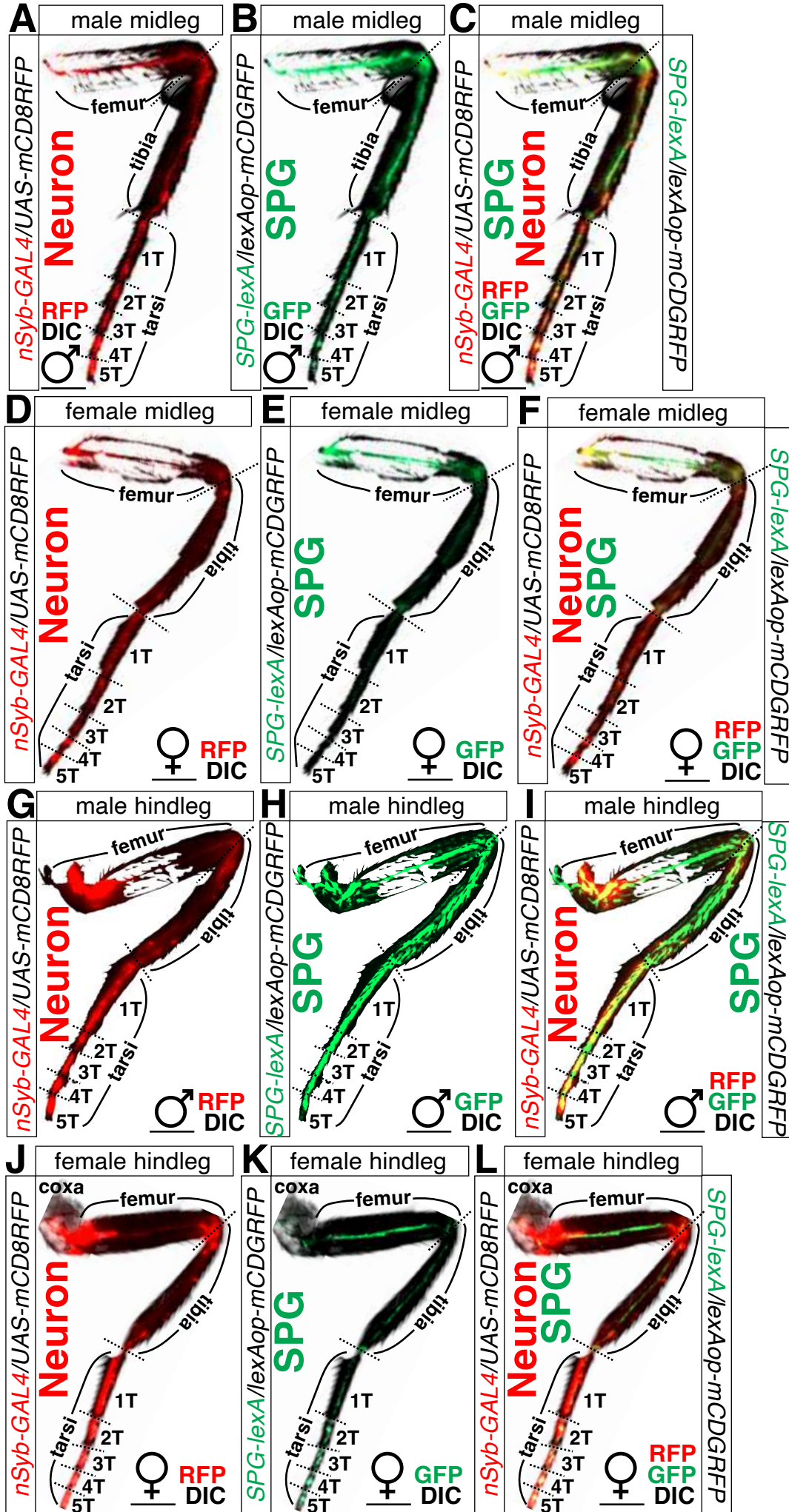

Supplement: Multimedia component 17 [file mmc17.pdf]

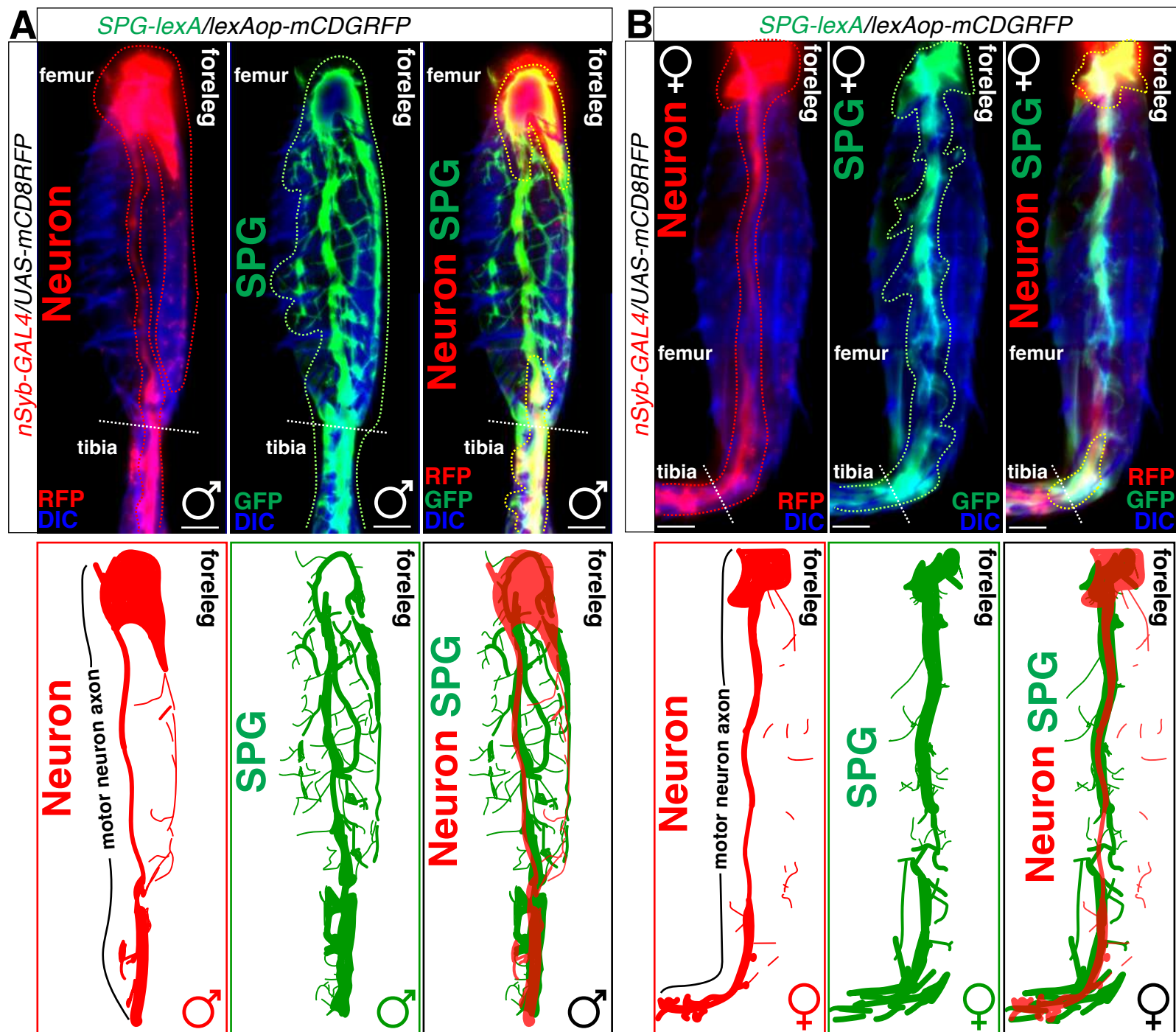

Supplement: Multimedia component 18 [file mmc18.pdf]

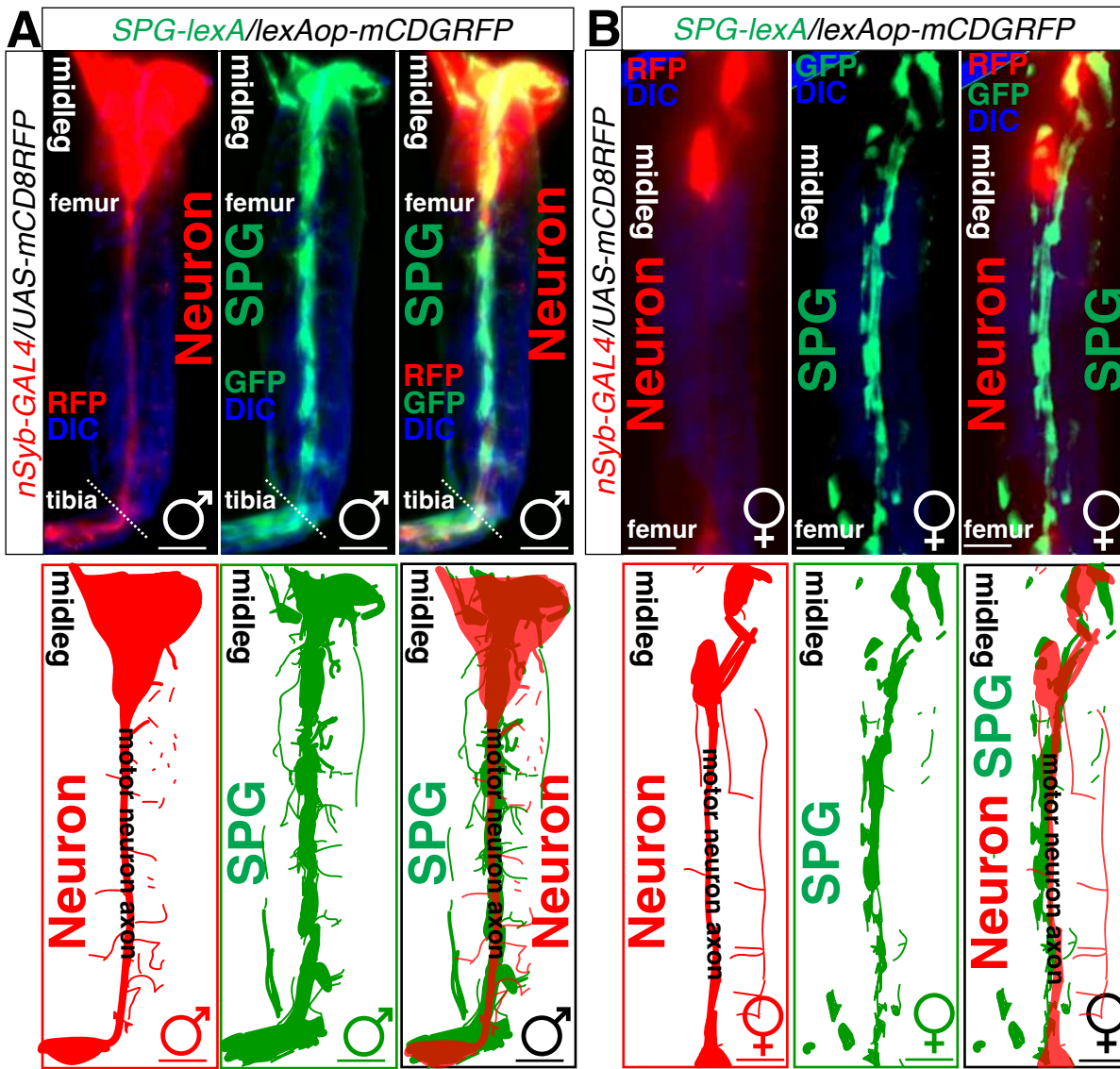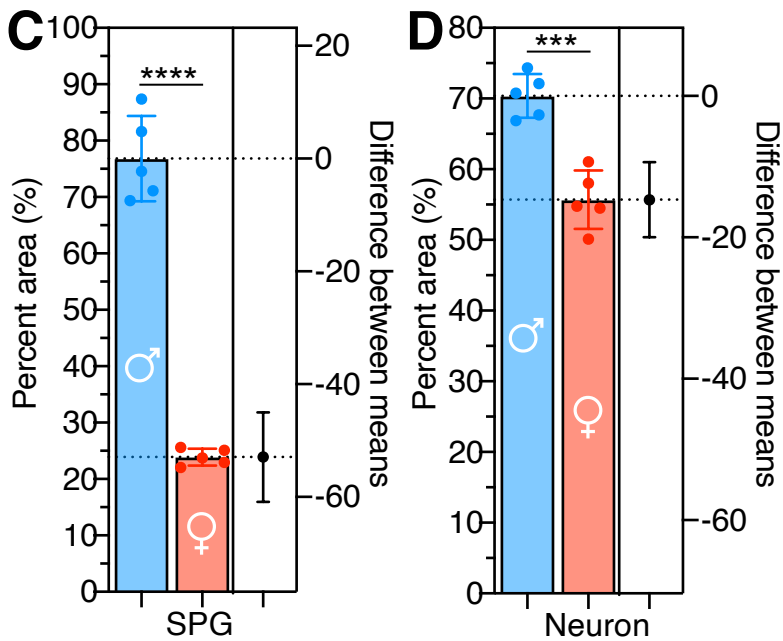

Supplement: Multimedia component 19 [file mmc19.pdf]

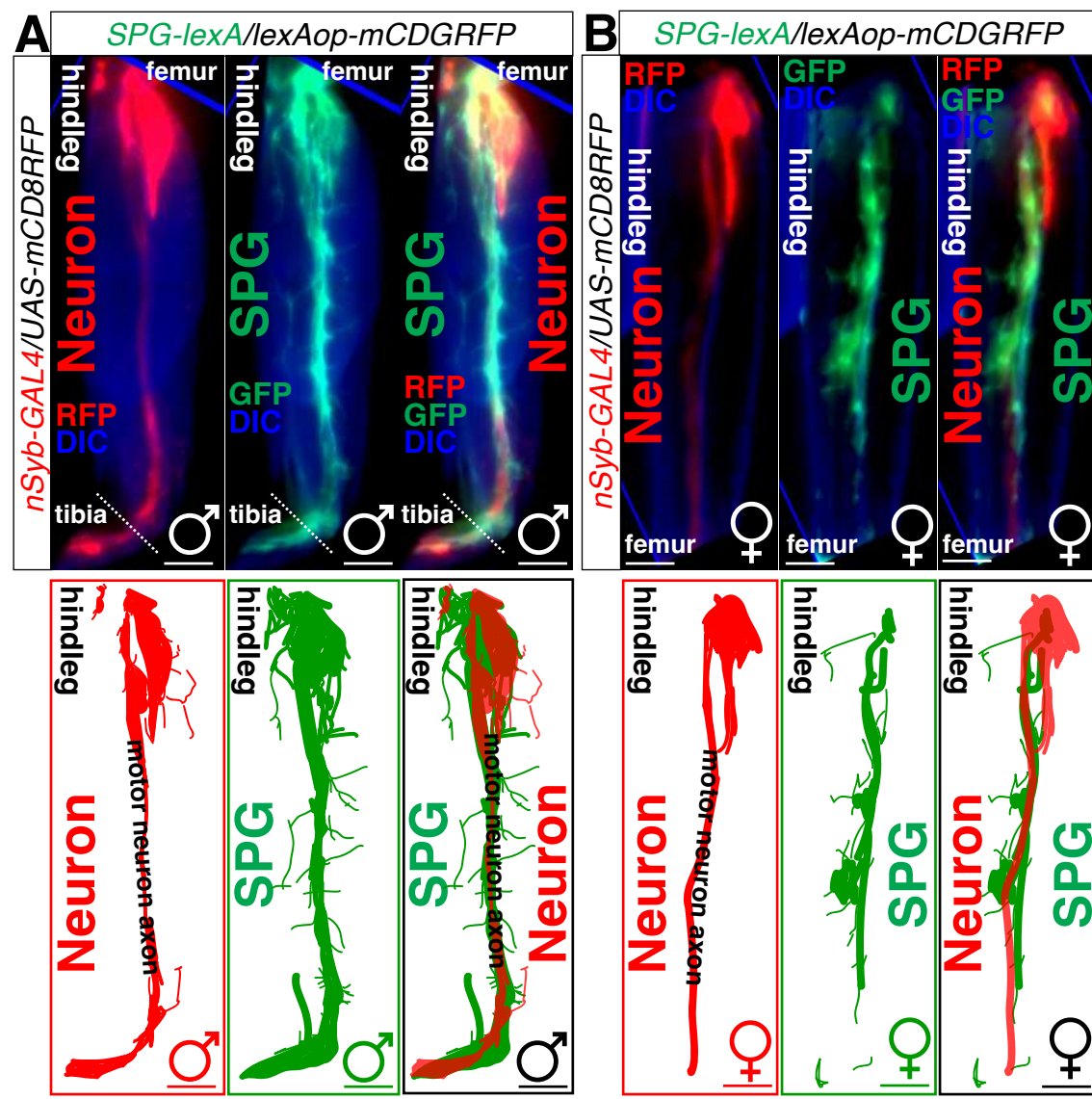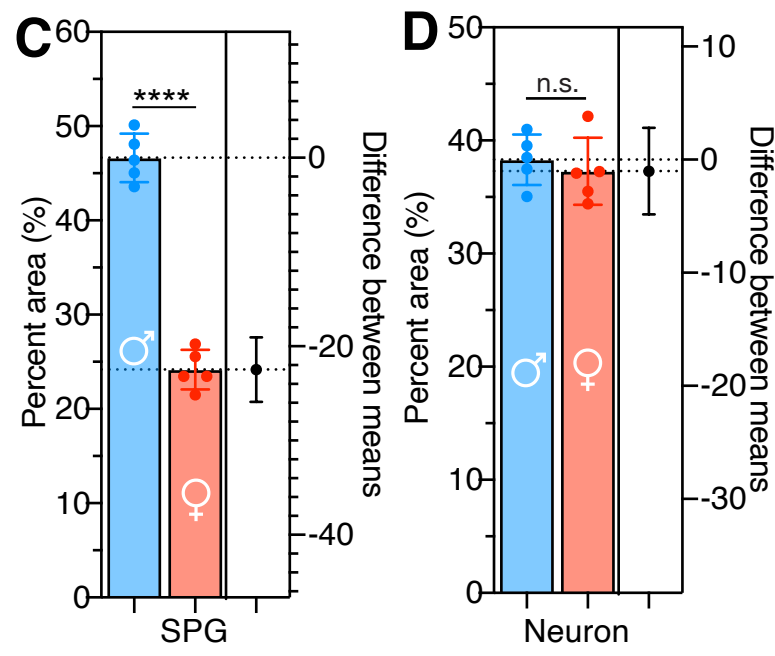

Supplement: Multimedia component 20 [file mmc20.pdf]

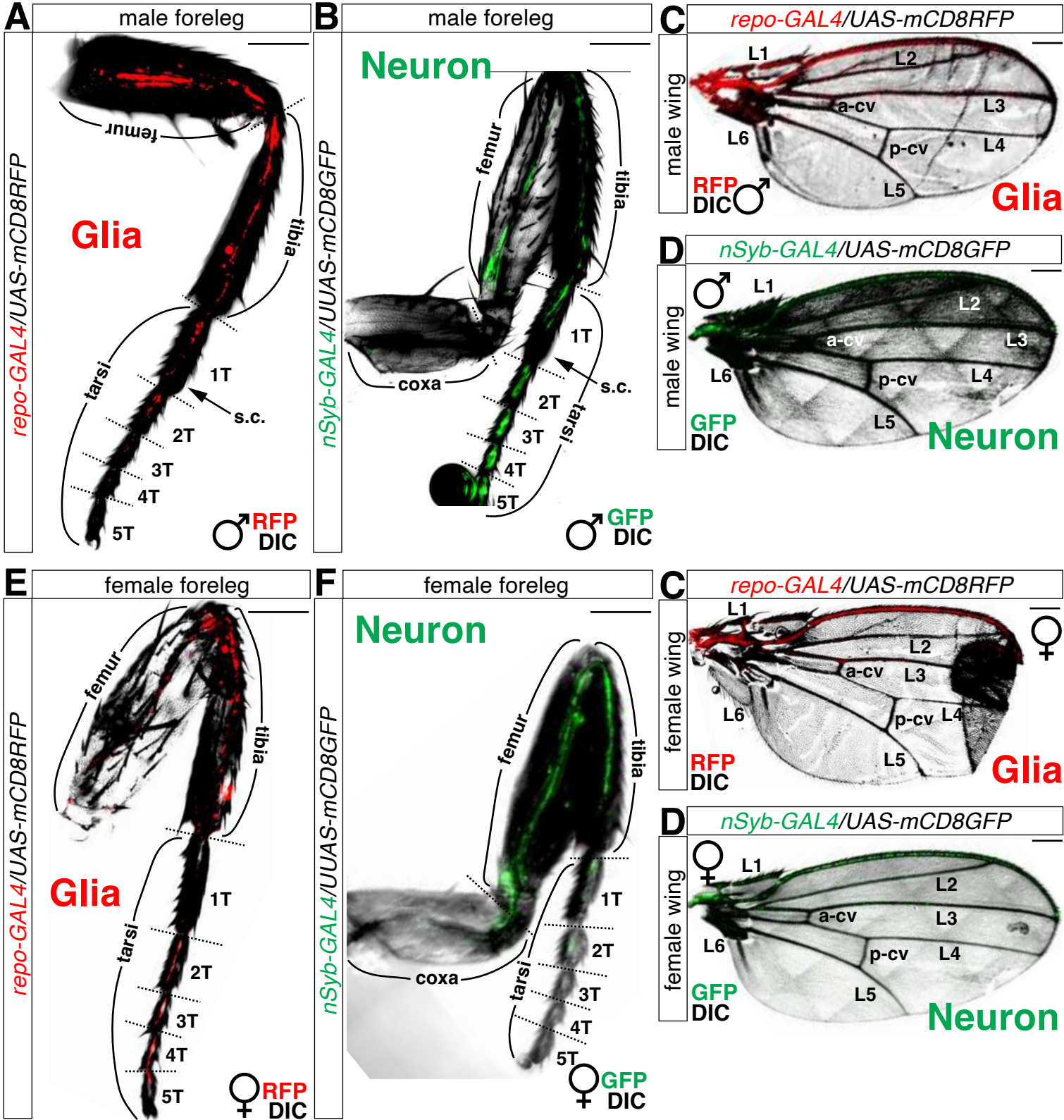

Supplement: Multimedia component 21 [file mmc21.pdf]
