# Supplementary material for: Glial subtype-specific modulation of disease pathogenesis in Drosophila models of ALS
Source: Genes Dis. 2025 Apr 8;12(5):101631. doi: 10.1016/j.gendis.2025.101631 (PMC12164006; doi:10.1016/j.gendis.2025.101631)
Supplement: Multimedia component 7 [file mmc7.pdf]

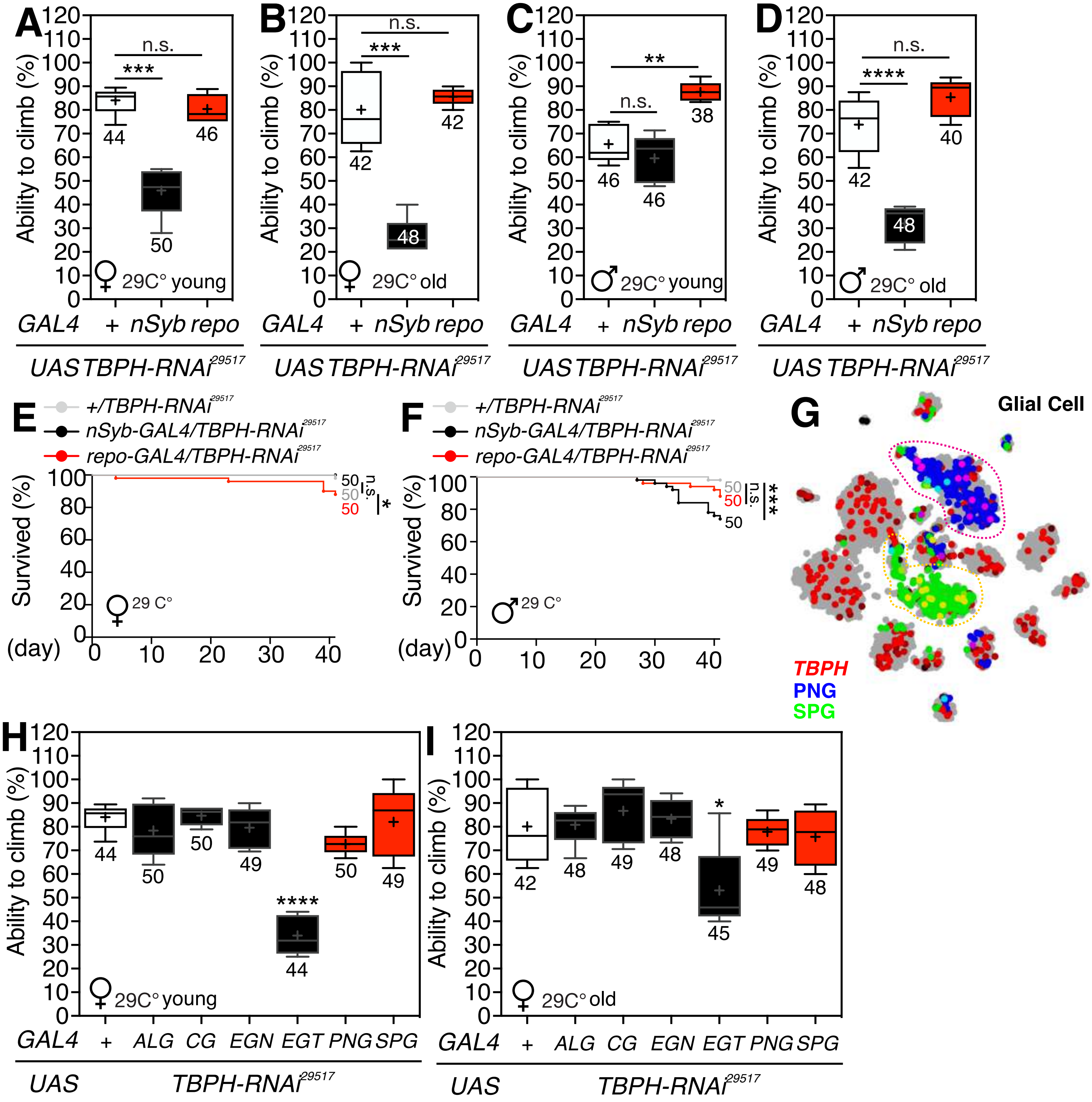

A
B
C
D
Ability to climb (%)
n.s.
***
**
****
29C° young
29C° old
GAL4 + nSyb repo
UAS TBPH-RNAi29517
E
F
+/TBPH-RNAi29517
nSyb-GAL4/TBPH-RNAi29517
repo-GAL4/TBPH-RNAi29517
Survived (%)
(day)
29 C°
G
Glial Cell
TBPH
PNG
SPG
H
I
GAL4 + ALG CG EGN EGT PNG SPG
UAS TBPH-RNAi29517

Wei&Snow SD.6
