# Supplementary material for: Glial subtype-specific modulation of disease pathogenesis in Drosophila models of ALS
Source: Genes Dis. 2025 Apr 8;12(5):101631. doi: 10.1016/j.gendis.2025.101631 (PMC12164006; doi:10.1016/j.gendis.2025.101631)
Supplement: Multimedia component 8 [file mmc8.pdf]

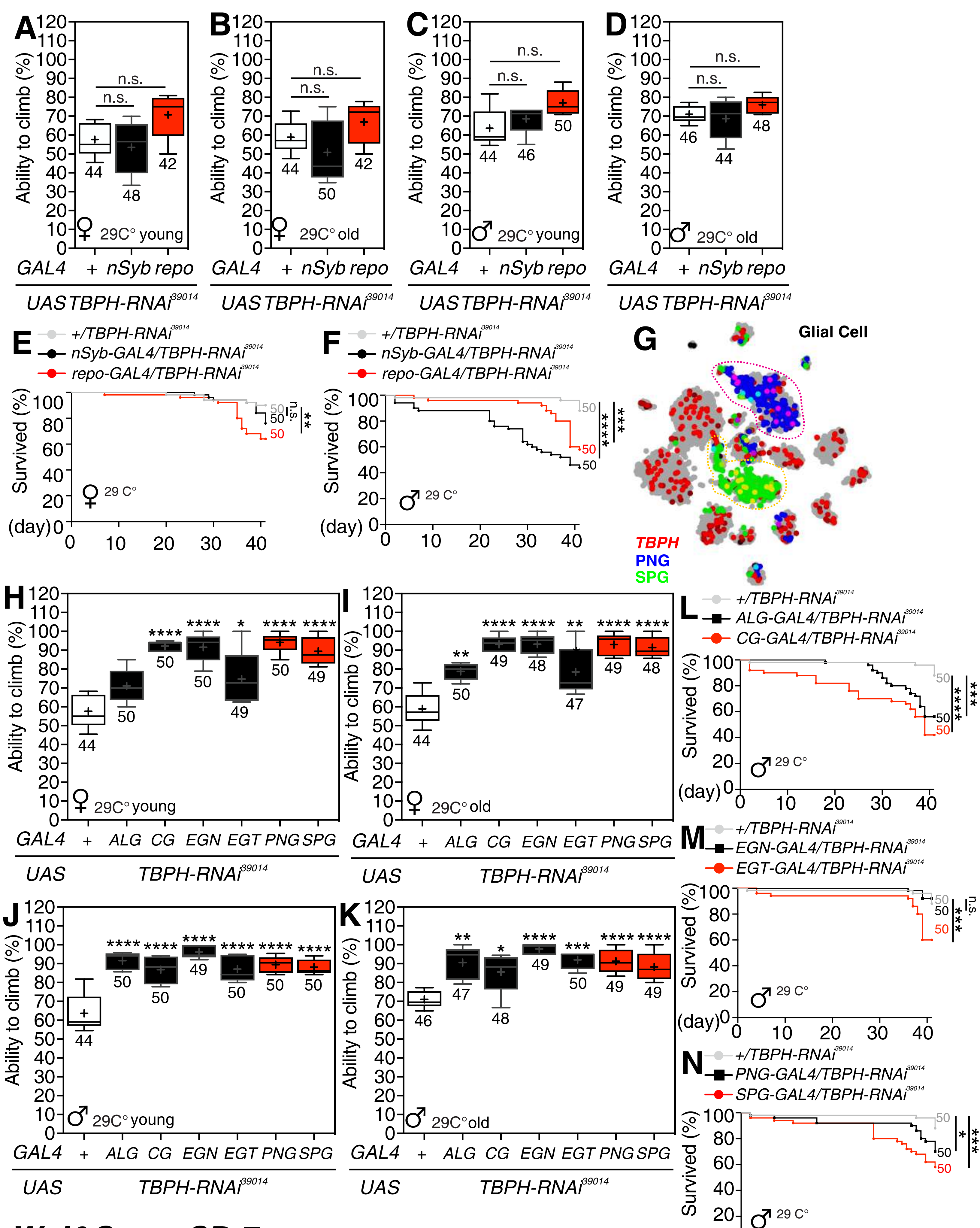

A
B
C
D
Ability to climb (%)
29C° young
29C° old
GAL4 + nSyb repo
UAS TBPH-RNAi39014
n.s.
E
F
G
+/TBPH-RNAi39014
nSyb-GAL4/TBPH-RNAi39014
repo-GAL4/TBPH-RNAi39014
Survived (%)
(day)
29 C°
Glial Cell
TBPH
PNG
SPG
H
I
J
K
GAL4 + ALG CG EGN EGT PNG SPG
UAS TBPH-RNAi39014
L
ALG-GAL4/TBPH-RNAi39014
CG-GAL4/TBPH-RNAi39014
M
EGN-GAL4/TBPH-RNAi39014
EGT-GAL4/TBPH-RNAi39014
N
PNG-GAL4/TBPH-RNAi39014
SPG-GAL4/TBPH-RNAi39014

Wei&Snow SD.7
